# A Census of Tandemly Repeated Polymorphic Loci in Genic Regions Through the Comparative Integration of Human Genome Assemblies

**DOI:** 10.3389/fgene.2018.00155

**Published:** 2018-05-02

**Authors:** Loredana M. Genovese, Filippo Geraci, Lucia Corrado, Eleonora Mangano, Romina D'Aurizio, Roberta Bordoni, Marco Severgnini, Giovanni Manzini, Gianluca De Bellis, Sandra D'Alfonso, Marco Pellegrini

**Affiliations:** ^1^Institute for Informatics and Telematics of CNR, Pisa, Italy; ^2^Department of Health Sciences, University of Eastern Piedmont Amedeo Avogadro, Novara, Italy; ^3^Institute for Biomedical Technologies of CNR, Segrate, Italy; ^4^Department of Science and Technological Innovation, University of Eastern Piedmont Amedeo Avogadro, Novara, Italy

**Keywords:** variable number tandem repeats, short tandem repeats, polymorphic tandem repeats, genic regions, catalog, tandem repeat detection tools, fuzzy tandem repeats, measure of polymorphism

## Abstract

Polymorphic Tandem Repeat (PTR) is a common form of polymorphism in the human genome. A PTR consists in a variation found in an individual (or in a population) of the number of repeating units of a Tandem Repeat (TR) locus of the genome with respect to the reference genome. Several phenotypic traits and diseases have been discovered to be strongly associated with or caused by specific PTR loci. PTR are further distinguished in two main classes: Short Tandem Repeats (STR) when the repeating unit has size up to 6 base pairs, and Variable Number Tandem Repeats (VNTR) for repeating units of size above 6 base pairs. As larger and larger populations are screened via high throughput sequencing projects, it becomes technically feasible and desirable to explore the association between PTR and a panoply of such traits and conditions. In order to facilitate these studies, we have devised a method for compiling catalogs of PTR from assembled genomes, and we have produced a catalog of PTR for genic regions (exons, introns, UTR and adjacent regions) of the human genome (GRCh38). We applied four different TR discovery software tools to uncover in the first phase 55,223,485 TR (after duplicate removal) in GRCh38, of which 373,173 were determined to be PTR in the second phase by comparison with five assembled human genomes. Of these, 263,266 are not included by state-of-the-art PTR catalogs. The new methodology is mainly based on a hierarchical and systematic application of alignment-based sequence comparisons to identify and measure the polymorphism of TR. While previous catalogs focus on the class of STR of small total size, we remove any size restrictions, aiming at the more general class of PTR, and we also target fuzzy TR by using specific detection tools. Similarly to other previous catalogs of human polymorphic loci, we focus our catalog toward applications in the discovery of disease-associated loci. Validation by cross-referencing with existing catalogs on common clinically-relevant loci shows good concordance. Overall, this proposed census of human PTR in genic regions is a shared resource (web accessible), complementary to existing catalogs, facilitating future genome-wide studies involving PTR.

## 1. Introduction

Tandem repeats (TR) in DNA sequences are patterns of similar subsequences directly adjacent to each other. The human genome is rich in TR, whose study is important for a wide range of applications in forensics, medical genetics, and population studies. A standard classification based on the number of bases in the repeating unit subdivides TR in microsatellites when the number of bases is within the range from 1 to 6 bps (for some authors from 1 to 10 bps), minisatellites for the range from 7 to 50 bps (for some authors the range stretches from 10 to 100 bps), and satellites when the number of bases in the repeating unit is beyond 50 bps (or beyond 100 bps). TR with a repeat unit from 1 to 10 Kb on a string are termed Tandem Copy Number Variations (TCNV) (He et al., 2011). In the scientific literature, microsatellites are also termed Short Tandem Repeats (STR), while minisatellites are also termed Variable Number Tandem Repeats (VNTR), when emphasis is placed on their highly polymorphic nature (Gelfand et al., 2014).

There are several important biological features that distinguish these classes, in particular VNTR vs. STR. The molecular mechanisms that generate variability of the number of repeating units of VNTR and STR loci in a population are distinct. In STR, repeat number variability is mostly generated by strand-slippage during replication by the DNA polymerase (Fan and Chu, 2007; Mirkin, 2007). In the case of VNTR, variability in the number of repeat units occurs mostly by events of unequal sister chromatid exchange (Wolff et al., 1991). Variability in biological samples at VNTR loci and STR loci is measured using different technologies. For microsatellite, variability detection is performed by PCR using primers directed to non-variable flanking regions, followed by fragment separation through electrophoresis (Edwards et al., 1991; Butler, 2007). Variability of VNTR is detected by restriction fragment length polymorphism (RFLP), a restriction digestion followed by Southern hybridization with a minisatellite probe (Nakamura et al., 1987; Sreenan et al., 1997).

Expansions in TR size are causative of more than 30 diseases, mostly neurodegenerative and neuromuscular disorders, including Huntington disease (HD), Kennedy disease (SBMA), and several types of Spinocerebral Ataxias (SCA) (Orr and Zoghbi, 2007). Since initially all known cases of TR-related diseases involved a repeating motif of 3 nucleotides, these diseases were denoted as trinucleotide repeat expansion diseases (TNR). However, cases of repeating units with 4, 5, and 12 nucleotides have been discovered (see Supplementary Materials), thus the more general notion of repeat expansion diseases (RE) became common. An interesting extreme case is that of the Prion Protein (PRPN), in which TR expansions with a unit size of 24 bps is associated with Creutzfeldt-Jakob disease (Goldfarb et al., 1991; Kovács et al., 2002). The TR involved in RE diseases thus fall mostly in the STR size range, although a few also fall in the VNTR range of motif size.

Typing VNTR, and STR is used for human population studies (Nakamura et al., 1987; Edwards et al., 1991; Pemberton et al., 2013; Putman and Carbone, 2014), for forensic applications (Lazaruk et al., 1998), for quantitative determination of allogenic bone marrow transplant engraftment (Schichman et al., 2002), and to differentiate sub-populations in studying the molecular epidemiology and phylogeny of bacteria (Smittipat et al., 2005). Variability in TR may also influence predisposition to cancer (Boland et al., 1998). PTR are a remarkable source of genetic variability since they display a wide range of values, different from the binary nature of single nucleotide polymorphism, allowing, in principle, a finer regulation of many different biological processes.

Large-scale analyses of STR in the human genome have been published in recent years (Payseur et al., 2011; Duitama et al., 2014; Willems et al., 2014), and interest has increased in the use of PTR (both STR and VNTR) for genomic studies involving human gene expression variations, complex traits, and complex diseases (Nakamura, 2009; Brookes, 2013; Gymrek et al., 2015).

For computational methods that scan long DNA sequences in order to detect TR, the size of the repeating unit, the sequence divergence among the single repeating units of a TR and the total size of the TR are the main features affecting their effectiveness. Many modern TR *in silico* detection methods are not so much affected by the differences between STR and VNTR, and indeed work equally well in both range of values, when the input is sufficiently long. For this reason we will refer more generically to the class of PTR, encompassing both STR and VNTR, when differentiating among them is not relevant for the discussion.

With the advent of high-throughput sequencing technology, in principle, it is now possible to sequence large cohorts of patients and search for highly variable TR in the sequenced data within genotype/phenotype association studies. However, highly repetitive regions and low entropy regions of the genome are technically difficult to sequence accurately with current Next Generation Sequencing (NGS) technologies, and even difficult to locate precisely onto the reference genome, thus the detection process is prone to high rates of false positives and false negatives. Overcoming these technical hurdles is a very active field of research. There is a pressing need for computational tools and resources supporting for this type of studies.

Data mining tools to detect polymorphism of TR loci in raw reads from sequencing assays are among the most useful supporting tools/resources. Examples of such data mining tools are: RepeatSeq (Highnam et al., 2012) lobSTR (Gymrek et al., 2012), ReviSTER (Tae et al., 2013), VNTRseek (Gelfand et al., 2014), myFLq (Van Neste et al., 2014). pSTR Finder (Lee et al., 2015), STR-FM (Fungtammasan et al., 2015), ExpansionHunter (Dolzhenko et al., 2016), HipSTR (Willems et al., 2017), and TREDPARSE (Tang et al., 2017). Recent surveys in Cao et al. (2014) and Gymrek (2017) compare the performance of many of these tools.

Catalogs of loci in the human genome, whose polymorphic nature is known (to a certain extent) and whose properties fit the purpose of subsequent analysis, are important as guides and facilitators for the tools used to detect PTR loci in raw reads. For example forensic studies usually refer to the Combined DNA Index System (CODIS) collection (O'Hara and O'Hara, 1956), while population studies make often use of the Marshfield polymorphic marker sets (McCarty et al., 2005). More recently Willems et al. (2014) produced a comprehensive landscape of variable STR in the human genome, based on data from the 1000 Genomes Project (Gymrek et al., 2015). Other catalogs have been produced by Duitama et al. (2014) by using the SERV predictor of polymorphism, and by Payseur et al. (2011) based on a direct comparison of two assembled genomes. These catalogs are suitable for studies at the genome-wide level.

In this article, we propose a new genome-wide catalog of PTR (which we refer to as the *census* throughout) and a general methodology to compile such catalogs starting from assembled genomes. Similarly to other previous catalogs of human polymorphic loci, we focused our catalog toward applications to the discovery of disease-associated loci. To this purpose, in order to ensure the presence in the catalogue of clinically relevant PTR loci, we have used a list of 37 clinically relevant PTR loci (see list in Supplementary Materials—Appendix A) as benchmark.

While many of the existing catalogs focus on the class of STR we aimed at supporting the detection of PTR with any motif size (within the technical limitations of the TR detection tools we employ). A recent survey of Gymrek (2017) mentions as an open problem that of extending the motif size of the TR considered in TR profiling studies beyond the standard range of STR motif size. Our work is a step in this direction. One of the key choices to attain this goal was to use as input assembled genomes (available at various stages of assembly: as scaffolds, or assembled chromosomes) from the NCBI web site.

## 2. Methods

### 2.1. Overview of the methods

#### 2.1.1. Main computational pipeline

**Figure 1:**
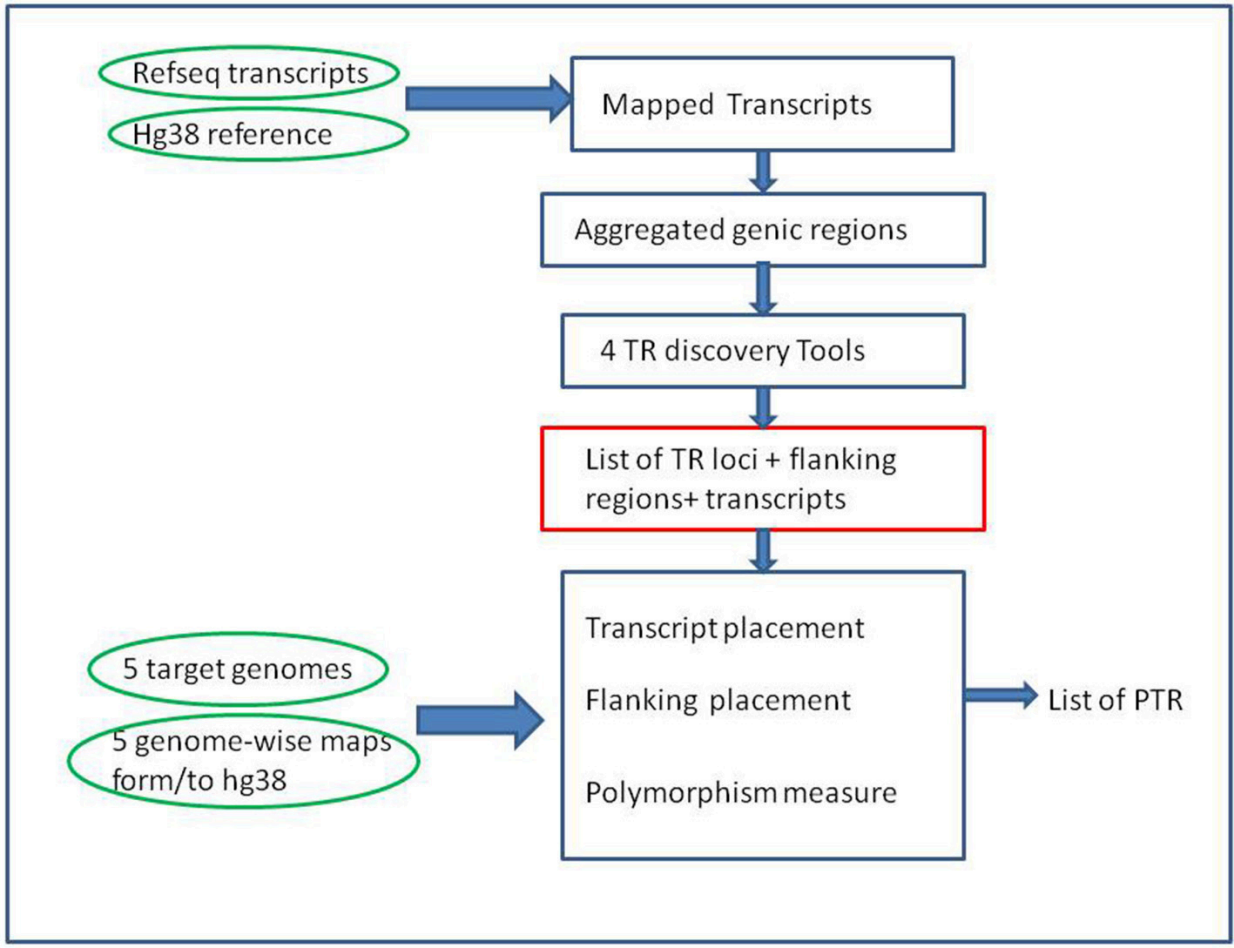
Overall computational pipeline. Green ovals represent the main input data sets, Blue rectangles computational steps, and the red rectangle a key intermediate result. Note that the TR discovery tools are applied only to the reference genome (GRCh38) in the initial part of the pipeline. The target genomes are used in the second part of the pipeline, as input for alignment-based procedures, to measure polymorphism of the candidate TR loci.

Building a catalog of PTR was done in two main phases (see Figure 1). The first phase was the compilation of a list of TR of interest by applying several existing and well established TR-detection software tools to the human reference genome. The second phase was the classification of each TR obtained in the previous phase as variable or non variable. For this second phase Willems et al. (2014) used the tool lobSTR (Gymrek et al., 2012) which was optimized for short-read data. Searching for PTR in short reads is a problem akin to searching them in long reads or in assembled genomes. However, the different quality of the input data and the limitations imposed by the short input sequences imply that methods oriented to short reads need to exert an effort to compensate for deficiencies in their input data. For this reasons a new procedure, described in this paper (see sections 2.1.4 and 2.2.4), was developed to classifying the variability of TR in our setting.

#### 2.1.2. Datasets

The reference genome used was the NCBI GRCh38 (Genome Reference Consortium Human Build 38). Five target genomes (in different stage of assembly) were downloaded from the NCBI portal, namely: (1) Venter's genome (huref assembly accession=GCF_000002125.1); (2) Chinese individual YH2.0 genome (assembly accession=GCA_000004845.2); (3) CHM1.1 genome (assembly accession=GCF_000306695.2); (4) African Individual BGIAF (assembly accession=GCA_000005465.1); and (5) ASM77258v3 genome (assembly accession=GCA_000772585.3).

The initial list of genes (transcripts) was downloaded from the UCSC data portal (RefSeq track). More details on the datasets used and on the preprocessing phases are in section 2.2. In particular our procedure for mapping transcripts to the target genomes in absence of a mapping provided by NCBI is described in the Supplementary Materials (Appendix B, C in Supplementary Material).

#### 2.1.3. Tools for TR detection and choice of parameters

We used four software tools to compile the intermediate list of candidate TR. The principal requirement was the availability of a stand-alone executable capable of processing long DNA sequences. The selected tools were TRF (tandem repeat finder) (Benson, 1999), mreps (Kolpakov et al., 2003), TandemSWAN (Boeva et al., 2006), and TRStalker (Pellegrini et al., 2010) (see Supplementary Materials—Appendix D for a brief description of each tool, and their URL).

The input sequences (obtained by merging overlapping transcripts) were extended 1,000 nucleotides in both directions, so that TR contiguous with the genic region could be identified. The parameters for the four tools were selected relying on previous studies in the literature. A dataset of 37 PTR reported to be disease-causing was used as a benchmark to evaluate the ability of the four tools to identify potentially relevant TR. Our experiments have shown that the four tools were able to detect all 37 TR with high precision (with Jaccard coefficient ≥ 0.7, data not shown).

#### 2.1.4. Procedure for measuring polymorphism

The procedure for measuring polymorphism of TR in the target genomes was based on three steps that are applied to each RefSeq transcript in turn, and to each TR included in the transcript under consideration. Step (1): the flanking regions of the model TR were identified in the corresponding transcript of the target genome. The sequence between the two flanking regions, if uniquely placed in the target, was termed the TR target. When the flanking sequences were not uniquely mapped, the encompassed TR was excluded from the downstream process. Step (2): the model TR and the target TR were tested for compatibility (e.g., it was tested whether one string is a substring of the other, allowing for a certain percentage of mismatches, insertions and deletions). Step (3): the number of repeat units was estimated. Within each phase, several quality scores were collected, filtering out candidates that did not pass the threshold set for each score. This pipeline ensured that the final list of candidate PTR was composed of high quality items.

The above pipeline can be seen as a generalization of the method adopted by Payseur et al. (2011), where, moreover, approximate and fuzzy TR are targeted, whereas Payseur et al. (2011) considered only pure TR. In the landscape catalog (Willems et al., 2014) the polymorphism was measured on a collection of short reads using the lobSTR tool (Gymrek et al., 2012). lobSTR was highly optimized for accepting as input short reads, and was based on a read indexing approach, as well as on a spectral analysis approach for identifying periodicity. Since our inputs are assembled genomes, read size issues were not constraining our choices and a different approach based on general purpose alignment tools of the BLAST family was adopted. Such BLAST tools allowed us to handle effectively higher error rates in several of the matching steps mentioned above.

#### 2.1.5. GO analysis

The GO analysis was conducted using R environment (v3.2.5). The biomaRT package v2.26.1 (bioconductor.org/packages/3.2/bioc/html/biomaRt.html) was exploited to retrieve the GO annotation (BP) of each gene using the hsapiens_gene_ensembl annotation (GRCh38.p10) of the ENSEMBL_MART_ENSEMBL database (Ensembl Genes 88). We used the hypergeometric test to check whether any biological function was enriched among the genes containing PTR. Benjamini-Hochberg and Bonferroni correction procedures were used to correct the raw p-value. Separate analyses were performed for PTR overlapping coding regions, exons, introns, UTR regions (5′ and 3′) and upstream (5′ and 3′), as well as the whole genes.

### 2.2. Methods in detail

#### 2.2.1. Compilation of the list of TR from the reference genome: input data preparation

A TR in which a motif is repeated identically many times (Pure TR) is easy to define and relatively easy to identify computationally within a sequence. However, when mutations (substitutions, insertions, deletions) are allowed between repeating units several different mathematical and statistical characterizations are possible, and this fact is also reflected in the variety of software tools at our disposal to find biologically significant repeats. In absence of a unique criterion to find and discriminate candidate TR, four software tools were used to compile our list of candidate TR: TRF (tandem repeat finder), Mreps, TandemSWAN, and TRStalker (see Appendix D for parameter details and a brief description of each tool). These tools were applied to the GRCh38 genome (accession number GCA_000001405.15) as a reference. Both the primary and alternative assemblies of the reference were scanned, extracting from them the sequences belonging to all known annotated RefSeq genes (downloaded from the UCSC table browser). In order to avoid multiple scanning of a sequence belonging to diverse isoforms of the same gene, overlapping sequences were merged prior to the tool application. However, sequences coding on opposite strands were kept separated. This choice was motivated from the fact that most of the TR discovery tools use the first instance of the motif sequence as a seed for extending the TR at a certain stage during the computation. As a result, the output of a given tool could change when a given sequence is scanned in the opposite direction. For example given the sequence ATT ATT ATC ATC ATT ATT ATC, and accepting only three mismatches from the consensus sequence, scanning the sequence from left to right the entire string is considered as a TR, while scanning it in the opposite direction the last unit of repeat would not be reported. After merging overlapping sequences, the number of input sequences was reduced from 82,960 to 28,144, without affecting the outcome and, in turn, a lower overall computational effort was required for the TR detection.

#### 2.2.2. Parameter tuning of TR detection tools

Parameter tuning is a delicate step in this type of applications, as one has to balance the resources for computation (time/storage) with the quality of the output (it is desirable to have a large number of high quality TR and a low number of low quality TR). We take advantage of insight from several comparative studies in the literature. The tool TRF has been extensively optimized by Gelfand et al. (2014) for VNTR detection and by Willems et al. (2014) for STR detection. We used the parameters selected by Willems et al. (2014) for TR with short period (≤6) (thus, within the STR range of values). In particular, this set of parameters (Willems et al., 2014) ensure a 1% False Discovery Rate (FDR) of the TRF tool applied to the reference genome with respect to a randomized genome obtained from a second order Markov chain. Gelfand et al. (2014) use a different (more relaxed) set of parameters for detecting candidate TR using TRF on the reference genome for motif size greater than 6 (thus, within the VNTR range of values). For mreps, TRStalker, and TandemSWAN, the default parameters described in the original publications were used, since they represent a good balance between execution time and output quality. For TRStalker and TandemSWAN such default parameters were sufficient to detect novel TR in comparative experiments (Boeva et al., 2006; Pellegrini et al., 2010). As a final check, the behavior of the four tools together with their chosen parameters was assessed on a collection of 37 clinically relevant PTR loci (see Supplementary Materials—Appendix A). All 37 loci were detected as TR in the reference genome (at a Jaccard coefficient 0.7, data not shown). Details of the parameters selected for each tool are in the Supplementary Materials—Appendix D.

Table 1 reports the raw number of TR discovered with each tool and the chosen parameters. These candidate TR were passed to the next phase aiming at annotating each TR with its polymorphism attribute (polymorphic/non polymorphic). For subsequent analysis, identical TR in the raw output from the 4 tools were identified and removed.

**Table 1:** Number of TR detected by each tool on GRCh38.

**Tool**	**Number of TR**
TRF	1.056.031
TRStalker	32.947.116
TandemSWAN	23.104.829
Mreps	3.097.654
Total	51.971.716

*Duplicate and quasi-duplicate removal is applied to the output list of each tools separately. The total is obtained re-applying the duplicate and quasi-duplicate removal procedure on the union of the four lists*.

#### 2.2.3. Location of TR in the target genomes

The polymorphism of TR was evaluated by means of an *ad-hoc* procedure: firstly, for each transcript and each TR of the list of TR resulting from the first phase contained in the transcript, the transcript was mapped on the target genome, and, if such mapping was unique, the flanking regions of the TR were mapped within this region. Then, the variability of the TR in terms of number of copies of the consensus motif of the TR was measured for each TR of each transcript in each target genome.

**Figure 2:**
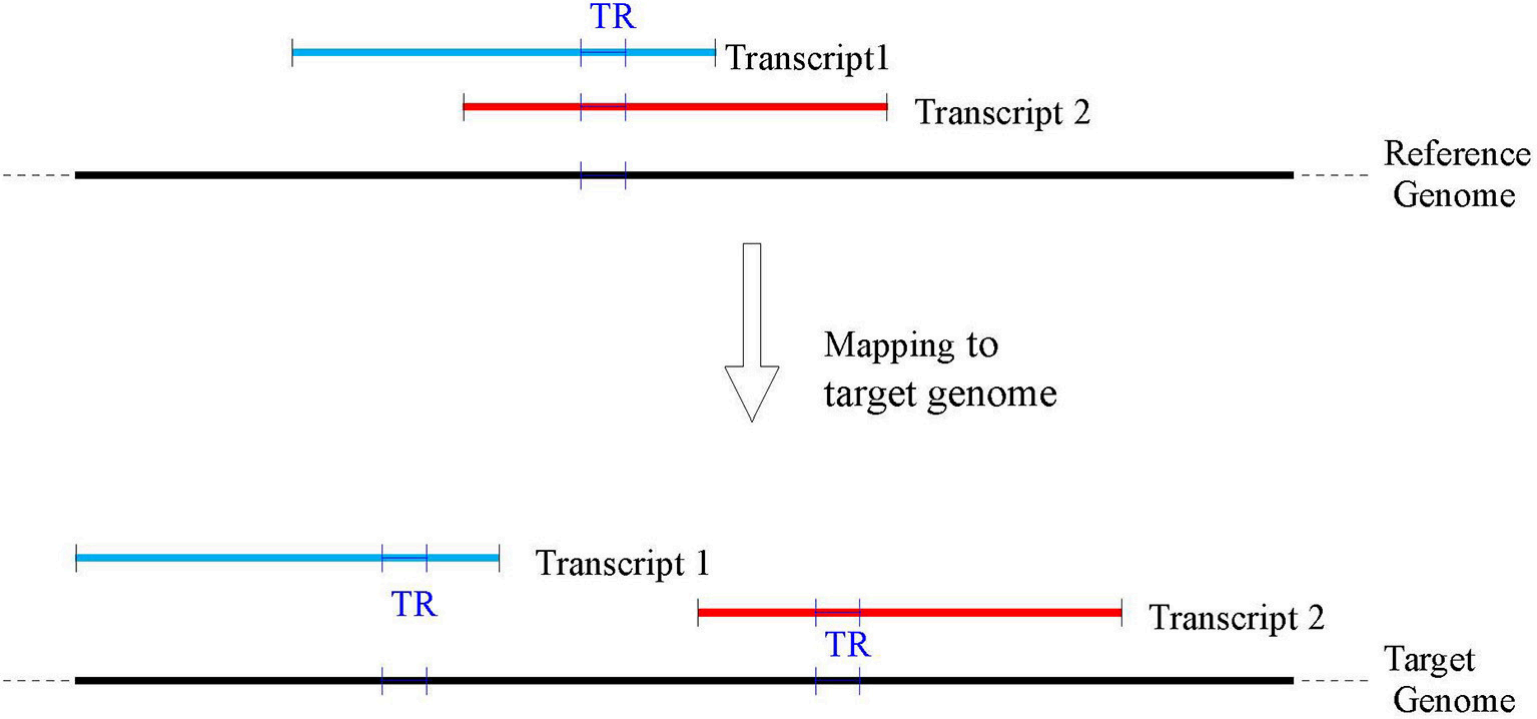
A TR may belong to several overlapping transcripts mapped in the reference genome (top of the figure), however as transcripts may map to non overlapping genomic locations in the target genomes (bottom of the figure), a single TR on the reference GRCh38 may be associated with several TR on each target genome.

A direct search of the TR sequence in the target genome is impractical for two main reasons: firstly TR sequences tend to be not unique along the genome, thus producing several equally probable locations; secondly the different number of copies between reference and target genomes due to potential polymorphism can produce a truncated placement (e.g., only a fraction of the TR from the reference is located in the target or vice versa). In order to overcome these problems, our TR location procedure is based on the alignment of the flanking regions of a TR to the corresponding gene in the target genome. Following the methodology of Payseur et al. (2011) flanking regions of size 250 bps were used. Wherever available (e.g., for Venter, YH2.0, and CHM1.1), the genic coordinates on the target genomes were extracted using the remapping service provided by NCBI (www.ncbi.nlm.nih.gov/genome/tools/remap). Subsequently, the corresponding sequences in the target genomes were extracted for further processing. Since at the time of this work the NCBI remapping service did not provide mapping for BGIAF and ASM77258v3, customized maps were built (see for details Supplementary materials—Appendix B and C). Because of a possible different rearrangement of the genes on each target genome, a given TR belonging to more than one gene in the reference could be mapped to multiple positions on a target genome (see Figure 2 for an illustration). Consequently, for each TR belonging to more than one transcript, the analysis was repeated on each mapped target sequence.

The target genes were extended to include the upstream and downstream regions. In addition, the boundaries of those sequences were extended with extra 250 bps to include possible flanking regions used to place TR located in the boundaries of the gene sequence. Finally, possible expansions of TR in the target genomes were taken into account. Because of a repeat expansion, a flanking region located at the boundary of the sequence could exceed the end of the target sequence causing the impossibility of placing a PTR. In order to avoid this case the boundaries of the target sequence were extended with extra 1,250 bps in each direction.

A careful choice of the size of the flanking regions is critical because such choice affects the identification of the TR. In fact, the shorter are the flanking regions, the higher is the probability of an incorrect placement. In contrast, flanking sequences that are too long could cause the placement procedure to fail because of local variations (such as SNP and short in/del) independently of the presence or absence of the TR. Following the methodology of Payseur et al. (2011), the size for the flanking regions was set at 250 bps. During the execution, this value ensured the placement of the large majority of the TR.

To increase the reliability of the placement procedure, the alignment of the flanking regions was performed only on the sequence in the target genome corresponding to the gene of the reference genome hosting the TR. Moreover, potentially ambiguous mappings were discarded when: 1) only one of the flanking regions aligned with sufficiently high identity score; or 2) the two flanking regions did not map in the appropriate order; or 3) at least one flanking region had multiple possible correct mappings.

The NCBI version of the BLAST+ software was used to perform local alignments of the flanking regions in the target sequences. Alignments with identity score lower than 90% were filtered out because they could produce misleading placements. In addition, both flanking regions considered as a whole had to satisfy additional conditions. In particular, at least 450 out of 500 bases had to be matched in the alignment. The sequence of each TR in each target genome was the output of this step (identification phase).

#### 2.2.4. Evaluation of polymorphism

The evaluation of the polymorphism index of a TR required the measurement of the expansion or contraction of a TR in terms of the number of repeat units in the target genomes relative to the reference genome.

As shown in Figure 2A TR on the reference genome can map to different positions over any of the target genomes (this phenomenon was due to the mapping of the RefSeq transcripts hosting a TR). Thus, for each TR, all the possible target locations of its RefSeq transcripts were tested and the TR was labeled polymorphic if a different number of repeat units was found for at least one mapped transcript. In order to provide a single high quality measure of the polymorphism for a TR, the target locations were first sorted according to a measure of quality of the polymorphism and according to the decreasing absolute difference of number of repeat units. The polymorphism value of the TR in the top scoring target transcript according to this ranking was returned. For further processing the number of such transcript target locations, and the polymorphism of each TR in them were recorded.

The measure of the polymorphism of two (source, target) TR was computed as follows. Let *S* = *s*_1_*s*_2_…*s*_*n*_ be the sequence of the TR on the reference and let *T* = *t*_1_*t*_2_…*t*_*m*_ be its corresponding sequence in a target genome (e.g., the region in the target genome between two mapped flanking sequences). The substring *s*_*i*_…*s*_*j*_ is denoted as *S*(*i, j*) (similarly for *T*). Suppose furthermore, without loss of generality, that *S* is shorter than *T*. Let *H*() be the evolutionary distance (Sellers, 1974), which is a generalization of the Hamming distance of two strings, and can be computed with a variant of the algorithm of Needleman and Wunsch (Gusfield, 1997). In the case of strings of unequal size, the two strings are aligned on their leftmost symbol, and the longest string is truncated to the size of the smallest. Our polymorphism check is based on two tests.

Test 1: given a threshold *k* (specified below), it is required that *S* and *T*(1, *n*) be very similar according to *H*(), allowing only *k* mismatches. Formally, *H*(*S, T*(1, *n*)) ≤ *k*.

Test 2: given a threshold *k*′, it is required that *S* and the residual of *T*, *T*(*n* + 1, *m*) be very similar according to *H*(), allowing only *k*′ mismatches.

Test 1 ensured that the source TR and the target TR share the same sequence (or in other terms the shorter TR is a prefix of the longer one). Test 2 ensured that the residue of T had approximately the same motif of the source TR. The two thresholds *k* and *k*′ were chosen as a small fraction of the size of the smallest sequence. In particular the value *k* = ⌊0.1 · *n*⌋ and *k*′ = ⌊0.1 · min(*n, m* − *n* − 1)⌋ was set.

The above tests were computed by means of two *ad-hoc* local alignments. BLAST+ could not be used for this task since this software requires sequences to have a minimum size incompatible with the small size of some TR. An *ad-hoc* alignment software based on the original Needleman algorithm was implemented (using standard weights: +1 for matches, −1 for mismatches and gaps) This software was rather memory intensive, therefore it has been optimized to evaluate both tests just using a single alignment matrix. Analyzing the whole catalog of TR in the target genomes required more than 5 billion alignments (performed either with BLAST+ and our Needleman implementation) therefore, a distributed pipeline was implemented to spread this computation among the CPUs of our computing cluster. This computation took anyhow a few weeks to be completed.

A naïve prediction of the variation of number of repeat units of a TR could be simply based on the difference between the size of the compared sequences. However, this approach could give an overestimation of the polymorphism, due to overcounting of gaps in the alignment.

**Figure 3:**
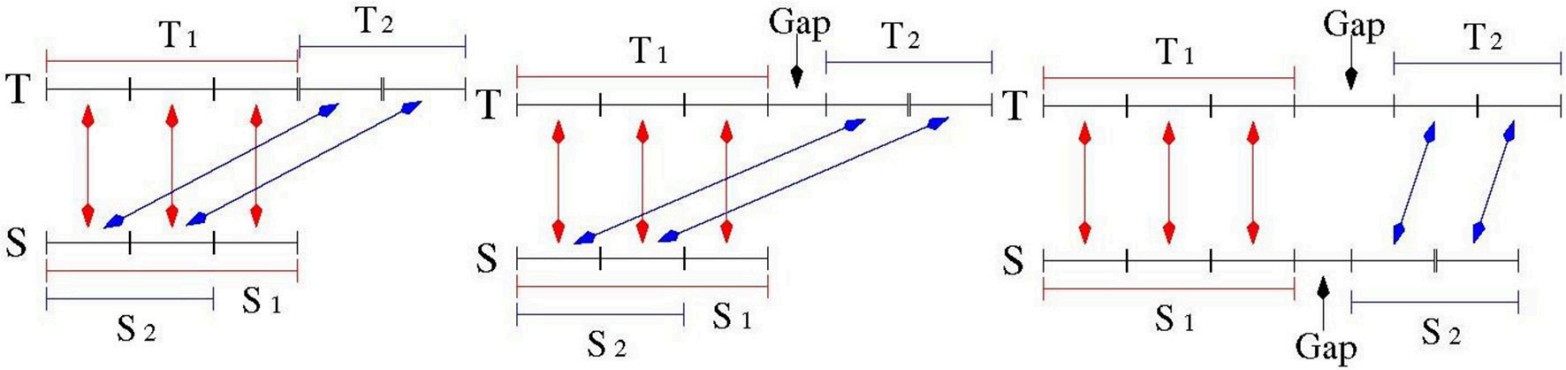
Quality Assessment for the measurement of TR expansion/contraction. Sequence *S* denotes a model TR in the reference genome, Sequence *T* denotes the corresponding TR in a target genome. Subsequence *T*_1_ matches *S*_1_ in the first alignment, subsequence *T*_2_ matches *S*_2_ in the second alignment. When *S*_2_ is a prefix of *S*_1_, and *T*_1_ is adjacent to *T*_2_: this is a *high quality* match (**Left** drawing). When *S*_2_ is a prefix of *S*_1_, and there is a small gap between *T*_1_ and *T*_2_: this is a *medium quality* match (**Middle** drawing). When there is a gap between *S*_1_ and *S*_2_, and there is a gap between *T*_1_ and *T*_2_: this is a *low quality* match (**Right** drawing).

The number of repeat units was computed as the (rounded) ratio of the number of matches in the two alignments of *S* and *T* divided by the average size of the motif of *S* (which was estimated by the TR detection tools). See Figure 3 for an illustration.

#### 2.2.5. Finishing the catalog

In this finishing phase, the measure of polymorphism computed by performing Test 1 and Test 2 was further analyzed to attach a quality score to such a measure (distinguishing high, medium and low quality classes).

In the ideal case, the two alignments of Test 1 and Test 2 over the sequence *S* should be highly overlapping. As illustrated in Figure 3A the portion of the string *S* involved in the alignment of Test 2 is a substring of the portion of *S* aligned in Test 1. Moreover, looking at the string *T*, the matched bases of the two alignments should lie adjacent to each other. This situation was then formalized as:

Test 3: let *S*_1_ and *S*_2_ be the substrings of *S* matched respectively in the first and second alignment with *T* according to Tests 1 and 2. Let *T*_1_ and *T*_2_ be the corresponding substrings on *T*. In order to break ambiguities in computing the substrings *S*_1_, *S*_2_, *T*_1_ and *T*_2_ the alignments having the highest matching score were selected and, in case of tie, the leftmost one was chosen. If *T*_1_ and *T*_2_ were adjacent and *S*_1_ and *S*_2_ were overlapping, then Test 3 was satisfied and the measure of polymorphism was classified as “high quality.”

Test 3 may fail for TR when small gaps between two consecutive motif sequences are allowed. In this case, the condition of Test 3 was relaxed by admitting that *T*_1_ and *T*_2_ may be separated by small gap (see Figure 3B). For TR with small motifs (up to 4 bps) and polymorphism expansion/contraction less than two units (namely: the second alignment was shorter than 8 bps), an additional property was required: the number of matches in the second alignment had to be more than half of the size of the shorter of the two aligned strings. TR satisfying this relaxed variant of condition 3 were classified as “medium quality.”

Figure 3C illustrates the case where *S*_1_ and *S*_2_ are disjoint. In this case, as well as in those failing Test 3, the TR measurement was classified as 'low quality', and this measurement was not used to compile the final catalog of PTR.

Low quality was also assigned in the following cases: PTR including a high number of unspecified nucleotides, (a stretch of at least 10 consecutive N); PTR derived from spurious results returned by the discovery tools; duplicates (e.g., two PTR predictions spanning the same nucleotides but with different motif size and/or number of repeat units parameters); PTR with motif size equal to 1.

Finally, PTR with a high degree of overlap were removed with an iterative procedure by Gelfand et al. (2014). Namely the procedure iteratively apply the following rule: if two TR overlap by more than 50% of their size, the shorter TR or, in case of tie by size, the TR with larger motif was removed.

#### 2.2.6. Handling segmental duplications

Segmental duplications are highly homologous duplicated sequences of the genome (of size ≥ 1Kbp) with identity above 90% (Bailey et al., 2002; Sharp et al., 2005), that overall make up about 5% of the human genome. Segmental duplications are an important source of ambiguity when the aim is obtaining unique placements of sequences onto a genome. Our computational pipeline took as input the RefSeq transcripts and mapped them to the GRCh38 reference genome in the initial part of the pipeline. Transcripts that did fall squarely in a region of segmental duplication in GRCh38 were likely to have multiple placements, therefore were discarded in this early phase. Similarly, when transcripts were mapped to target genomes, transcripts having multiple placements in a target genome were discarded. This ensured uniqueness of transcript placement. Similarly, when placing flanking regions within a mapped transcript such placements were required to be unique (otherwise, the TR was discarded either from the reference or the target genomes). The only remaining possible source of multiplicity is depicted in Figure 2, where uniquely mapped overlapping transcripts in the reference genome do not overlap in the target genomes. This might be due to genomic rearrangements between individual genomes, or to different assembly policies. Note, however, that the number of such multiple images was limited (at most, one per transcript per TR) and it was computationally feasible to analyze each one for measuring a possible polymorphism.

## 3. Results

### 3.1. Validation by cross-referencing with existing catalogs

In order to validate the final polymorphism prediction, the census was compared with data from two large population studies profiled with lobSTR (Gymrek et al., 2012), on the set of disease-related loci. Results on the application of lobSTR to data from the 1000 Genomes Project (phase 1) (Willems et al., 2014) and from the Simons Genome Diversity Project (SGDP) (Mallick et al., 2016) were used (downloaded from http://strcat.teamerlich.org/download). The two collections contain the genotype of 1009 individuals sampled form 26 diverse populations (1KGP-phase 1) and 300 individuals sampled form 142 diverse populations (SGDP). Note that a direct application of our pipeline to such data was not possible, since its content is made up of unassembled (paired) short reads. Thus, our validation strategy was based on assessing whether the polymorphism measured in 5 assembled genomes was present also in some samples of the two collections. This methodology implies reliance on lobSTR as a correct tool to profile STR from reads. In support of this assumption, Mallick et al. (2016) reported a good concordance (*r*^2^ = 0.92) between capillary sequencing calls and lobSTR calls for 127 loci.

Table 2 reports, for 22 diseases (23 loci), 45 polymorphisms measured in the census data, distinguishing expansion (positive) of contractions (negative) in bp, relative to the reference genome. The column 'data' reports whether the data set used for the comparison (1KGP or SGDP) is informative on each locus. A data set is not informative if it has no measurement for the locus, or if measurements were reported only for less than 5% of the sampled population. The column 'in range' reports whether the predicted polymorphic size in the census is within the interval of values present in either of the data sets (1KGP or SGDP). The column “samples” reports, for loci sufficiently informative and within range, the number of samples that exhibit the same value as measured in the census in at least one of the two alleles. Raw data at individual level are provided as Supplementary Table.

**Table 2:** Cross-referencing of measured polymorphism at disease loci for the census data.

	**Census**			**1KGP**			**SGDP**		
**No**.	**Disease**	**pos**.	**poly (bp)**	**Data**	**In range**	**Samples**	**Data**	**In range**	**Samples**
1	Schizophrenia	chr1	+12	Y	Y	6	Y	Y	20
2			+5	Y	Y	(^#^)0	Y	Y	(^#^)0
3			(^**^)+45	Y	N	−	Y	N	−
4	DM2/PROMM	chr3	−18	Y	Y	0	Y	Y	2
5			−28	Y	Y	0	Y	N	−
6	Huntington (HD)	chr4	−3	N	−	−	Y	Y	3
7			+12	N	−	−	Y	Y	2
8	SCA12	chr5	(^**^)(^*^) +79	Y	N	−	Y	N	−
9	SCA1	chr6	+12	N	−	−	N	−	−
10			+13	N	−	−	N	−	−
11	SCA17	chr6	−6	N	−	−	N	−	−
12			+3	N	−	−	N	−	−
13	ALS−FTD 2	chr9	+6	N	−	−	Y	Y	1
14			−3	N	−	−	Y	Y	1
15			(^**^)+47	N	−	−	Y	N	−
16	Friedreich Ataxia	chr9	+5	N	−	−	Y	Y	4
17			+6	N	−	−	Y	Y	21
18			+11	N	−	−	Y	Y	20
19	DRPLA	chr12	−2	Y	Y	(^#^)0	Y	Y	(^#^)0
20			−15	Y	Y	57	Y	Y	58
21			+3	Y	Y	13	Y	Y	33
22	CCHS	chr12	+3	N	−	−	Y	Y	11
23	SCA2	chr12	−3	N	−	−	Y	Y	33
24	SCA8	chr13	−6	N	−	−	Y	Y	41
25	holomprosencephaly	chr13	−20	N	−	−	N	−	−
26	SCA3	chr14	+30	Y	Y	5	Y	N	−
27			+27	Y	Y	24	Y	N	−
28	Huntington 2 (HDL2)	chr16	+6	Y	Y	16	Y	Y	94
29			+9	Y	Y	3	Y	Y	9
30	Fuchs Corneal Dystrophy	chr18	+9	N	−	−	N	−	−
31			+12	N	−	−	N	−	−
32			−20	N	−	−	N	−	−
33			−39	N	−	−	N	−	−
34			−27	N	−	−	N	−	−
35	SCA6	chr19	−6	Y	Y	14	Y	Y	96
36			−18	Y	Y	8	Y	Y	45
36	miotonic dystrophy	chr19	−27	Y	Y	30	Y	Y	6
38			−45	Y	Y	228	Y	N	−
39	SCA36	chr20	+6	N	−	−	Y	Y	15
40			+12	N	−	−	Y	Y	101
41	SCA10	chr 22	+5	Y	Y	5	Y	Y	33
42			+10	Y	Y	2	Y	Y	4
43	SBMA (Kennedy)	chrX	−9	N	−	−	N	−	−
44			−45	N	−	−	N	−	−
45	fragile X	chrX	+30	N	−	−	N	−	−

*For 22 diseases (23 loci), the measured polymorphism (+, expansion, −, contraction) in bp are compared with genotypes in the 1000 Genomes Project (1KGP) and in the Simon Genome Diversity project (SGDP) obtained by lobSTR. The column “data” (Y/N) reports whether the data set (1KGP or SGDP) is informative on each locus. The column “in range” (Y/N) reports whether the census prediction is within the interval of values present in the data set (1KGP or SGDP). In column “samples,” for loci sufficiently informative, it is reported the number of samples that exhibit the same value as measured in the census (1009 samples for 1KGP 300 for SGDP)*.

(*) *for the SCA12 locus the expansion is given by a stretch of N thus it is annotated as “low quality'*.

(**) *for the Schizophrenia, the SCA12 and the ALS-FTD2 polymorphism the total size of the expanded TR is above the limit of lobSTR detection (which is 80 bps, for reads of 100s bps)*.

(#) *for Shizoprenia and DRPLA there are alleles in the 1KGP and SGDP data within 1bp of the measured census polymorphism*.

For 3 cases (related to Schizophrenia, SCA12 and ALS-FTD2) a polymorphism measured in the census was beyond the range of valid values of the lobSTR tool (for reads of size 100 bps, the largest detectable STR for lobSTR was of size 80 bps). For 13 cases neither the 1KGP nor the SGDP data set were informative. Thus, these 16 cases were excluded from further comparisons. For the remaining 29 cases, in 26 it was verified the presence of samples in the SGDP or 1KGP data sets with an identical polymorphism, in at least one of the two alleles. For two of the remaining 3 cases the predicted polymorphism size was within 1bp of a polymorphism in the SGDP or 1KGP data sets. For one case the measured polymorphism was within the interval between the smallest and the largest expansion/contraction present in the SGDP or 1KGP data sets. Overall, in 28 cases out of 29 the measured polymorphism is highly (or moderately) compatible with 1KGP and SGDP data (96.5%). For one case (3.5%) the measured polymorphism was not incompatible with 1KGP and SGDP data. In all other cases (16) the comparison was not possible.

### 3.2. Contribution of tools and genomes to the PTR catalog

**Figure 4:**
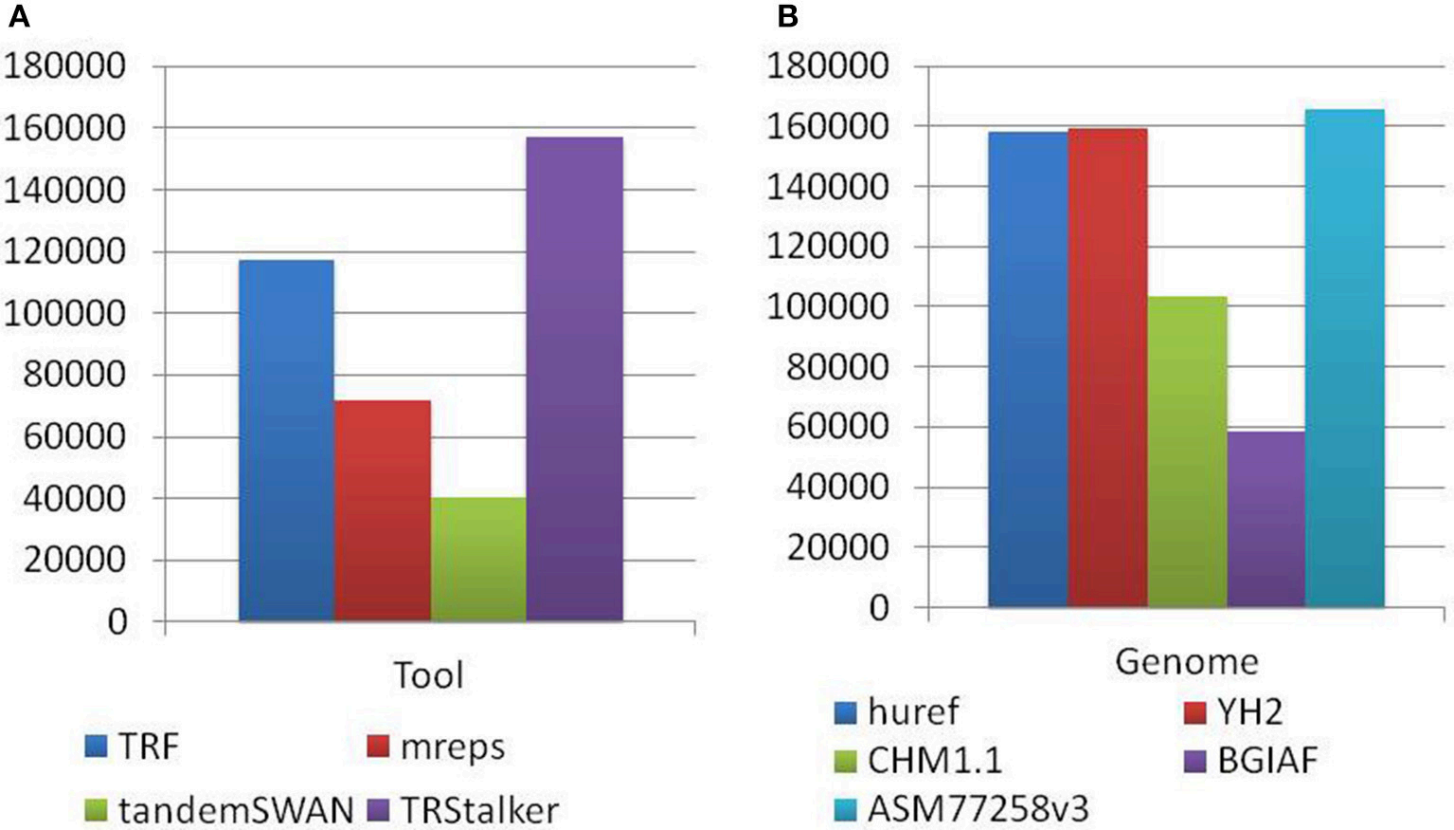
Distribution of PTR in the census relative to the computational pipeline. In the discovery pipeline the initial part of the pipeline produces a list of candidate TR by using four TR discovery tools. In the second part these candidates are classified as polymorphic (thus included in the output PTR listing) or not polymorphic by using five target genomes. **(A)** Numbers obtained by tracing back each PTR locus to the tool (o tools) that discovered the associated TR candidate. **(B)** Numbers obtained by tracing back each PTR locus to the target genome (or genomes) giving evidence that led to to classifying it as polymorphic. Note that each PTR may be counted in more than one column of subfigures **(A,B)**.

Each PTR in the final catalogue can be traced back to a TR (of the reference genome) discovered by one or more TR discovery tools, and also to a polymorphism detected on one or more target genomes. Note that a PTR may be associated with a TR detected by more than one tool and can be polymorphic in more than one target genome. The first basic statistics reports the separate impact of each tool and each target genome in the final listing of PTR. Figure 4A reports for each discovery tool the raw number of PTR that are associated with TR discovered by that tool. TRStalker ranks first in the number of PTR associated with a TR, followed by TRF, mreps, and TandemSWAN. Figure 4B reports for each target genome, the raw number of PTR that are associated with a polymorphism detected in that target genome. Three genomes (huref, YH2, and ASM77258v3) contribute an equal number of PTR (about 160,000) followed by CHM1.1 (about 100,000) and BGIAF (about 60,000).

**Figure 5:**
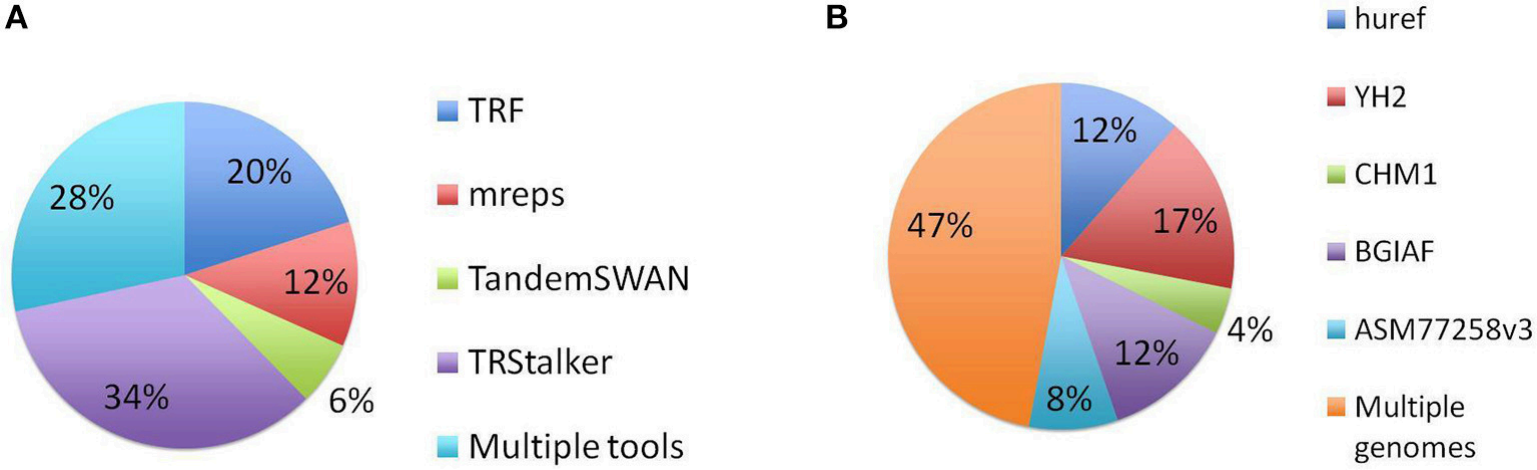
Distribution of PTR in the census relative to the computational pipeline. In the discovery pipeline the initial part of the pipeline produces a list of candidate TR by using four TR discovery tools. In the second part these candidates are classified as polymorphic (thus included in the PTR listing) or not polymorphic by using five target genomes. **(A)** Numbers obtained by tracing back each PTR locus to the *single* tool that discovered the associated TR candidate, or to multiple tools. **(B)** Numbers obtained by tracing back each PTR locus to the *single* target genome giving evidence that led to to classifying it as polymorphic or to multiple genomes. The identification of two TR is done with Jaccard coefficient threshold *j* = 0.7. Note that in the pie charts **(A,B)** each PTR is counted in one and only one category.

A more refined analysis discriminates PTR that are traced back to a TR detected by a single discovery software, and those that are associated with a TR discovered by two or more such tools. The implication is that a PTR associated with a TR discovered by a single software would be missed in a computational setting in which such tool is not applied. This analysis in retrospect validates the design decision of using multiple TR detection tools. This analysis is based on a notion of matching applied to the PTR annotated with their detection information collected along the computational pipeline. For this task the Jaccard coefficient applied to the genomic coordinates of the PTR (on GRCh38) was used^1^. Two TR from different tools were considered identical if their Jaccard coefficient exceeded the threshold value *j*. In Figure 5A is given the result of this analysis for *j* = 0.7. For this value, about 28% of the catalog can be traced back to TR detected by more than one tool. Using higher thresholds values (*j* = 0.9, 1.0) the fraction of PTR associated with TR that are matched shrinks to 10 and 3%, respectively, while using lower threshold (*j* = 0.5), this percentage increases to 41%. The complementary percentage of PTR associated with a TR detected by only one of the four tools ranges from 97 to 59%, depending on the threshold *j* (data not shown).

Similarly, for the target genomes Figure 5B gives the number of PTR associated with polymorphisms detected in multiple target genomes and those associated with polymorphisms detected a single genome among the five considered in this study, when the Jaccard threshold is set to 0.7. In this case, varying the Jaccard threshold from 1.0 to 0.5 has minimal influence on this distribution. About 50% of the loci are polymorphic in at least two target genome assemblies. Also, interestingly, the BGIAF target genome, which contributes the least in total number of PTR (see Figure 4), has the second largest number of PTR whose polymorphism is measured only in the BGIAF target genome.

### 3.3. Distribution of PTR in the census: basic statistics

**Figure 6:**
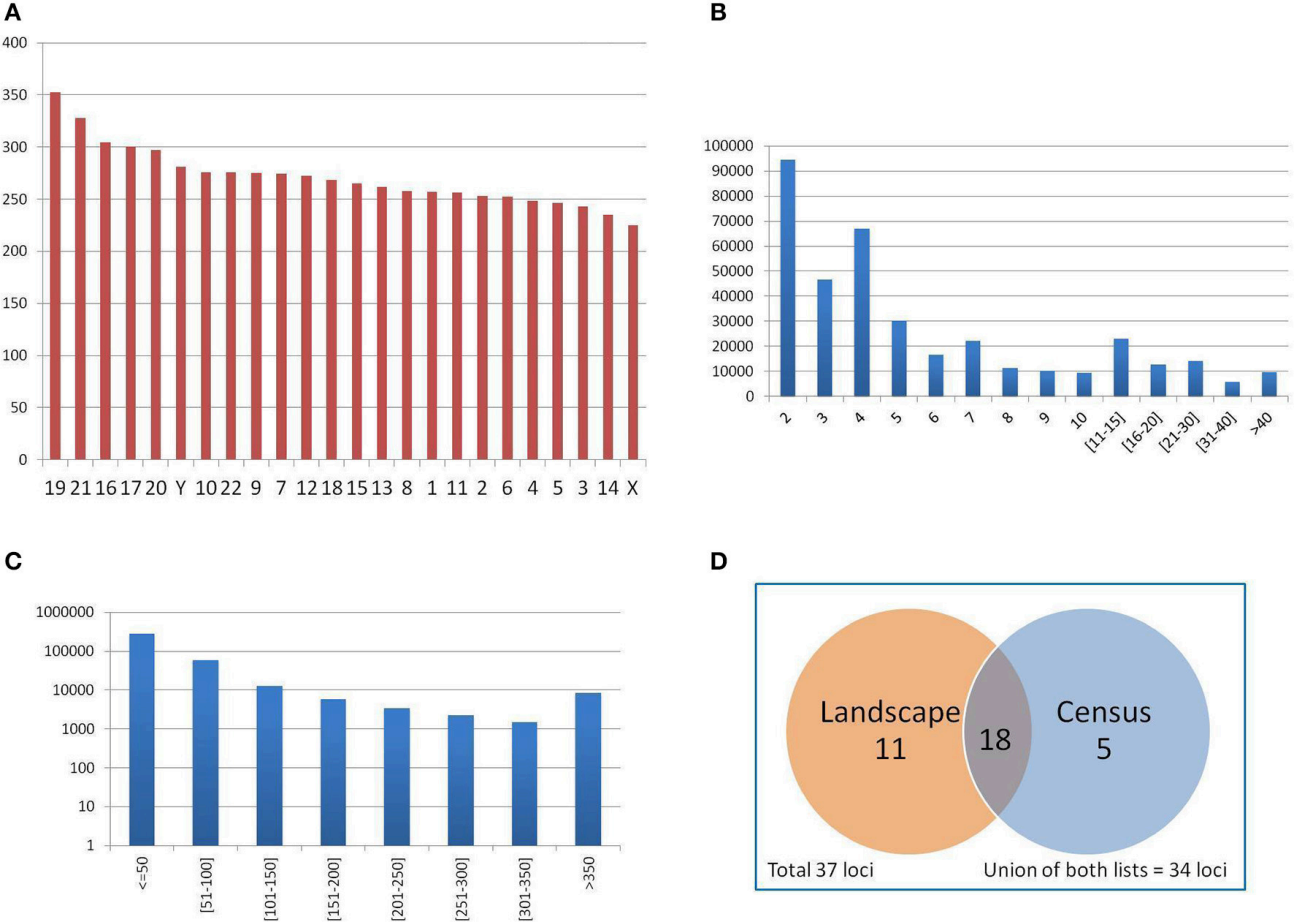
**(A)** Average number of PTR per Mbp of genic regions (merging of gene transcripts) in each chromosome. Autosomal chromosomes are numbered from 1 to 22, sexual chromosomes X and Y. Chromosomes are sorted in decreasing PTR density values. **(B)** Distribution of PTR per number of repeating units (in the reference genome). **(C)** Distribution of PTR per total size of the PTR loci (in the reference genome), in logarithmic scale. **(D)** Classification of 37 clinically relevant loci as PTR in the census and landscape catalogs.

The mean number of PTR per Mbp of genic regions analyzed in each chromosome (such regions include exons, introns, UTR and upstream, downstream sub-regions) was computed, in order to assess the uniformity of PTR detection over the gene regions in human chromosomes. The results for individual chromosomes are shown in Figure 6A. The mean PTR density per Mbp was 271.15 (std. dev. 28.9), with a maximum of 352 (chr 19) and a minimum of 224 (chr X). Thus, the mean density of PTR loci in genic regions in each chromosome had values within a rather narrow range (with about 10% standard deviation).

The distribution of PTR by their number of repeat units in the reference genome is reported in Figure 6B. Data on single values in the range from 2 to 10 showed a steady decrease, with two notable drops for values 4 and 6. For the next two intervals of size 5 (from 11 to 15, and from 16 to 20) there was also a decrease (in aggregation), as well as in two size 10 intervals (form 21 to 30 and from 31 to 40).

The distribution of PTR by total size in bp is shown in Figure 6C (in logarithmic scale). The vast majority of PTR (order of 10^5^) is in the range of size less than 50 bps. There is then an exponential decrease in the next two intervals (for a size in the range of 50–100 bps, the count is of the order of 10^4^, while for the range from 100 to 150 bps, it is of the order of 10^3^). For intervals of 50 bp from 150 to 350 bps, the count is of the order of 10^3^ per interval. The long tail of ≥ 350 bps overall counts about 10^4^ PTR. This size stratification is important since populating the catalog with PTR of large size was one of the aims guiding the design of the computational pipeline. Moreover, one can assess directly from this distribution the fraction of the PTR in the catalog that can be captured in an individual when a specific sequencing technology is employed, based on read length and on the possible size of the PTR expansions.

Of the 37 clinically relevant loci (Supplementary Materials—Appendix E) 23 are classified in the census data as polymorphic (62%) (see Figure 6D). The landscape catalog (Willems et al., 2014) classifies 29 loci of the 37 clinically relevant loci (78%) as polymorphic. Comparing the single loci of the census and the landscape catalogs, 18 loci are common to both lists, 5 loci present in the census are not detected in the landscape, while 11 loci in the landscape are not detected in the census data. The union of the two catalogs classifies correctly 34 over 37 clinically relevant loci (92%) as PTR. Figure 6D summarizes the classification of clinically relevant loci in the census and landscape catalogs.

### 3.4. Distribution of PTR/TR ratio by motif size and number of repeat units

Payseur et al. (2011) note a strong positive dependence of the proportion of variable STR loci (over the total number of loci), with the number of repeating units (in the reference genome), and notice a steep increase in variability in the range of repeating units from 5 to 12, and a plateau for higher values (Payseur et al., 2011, Figure 1). The influence of the size of the repeating motif is also analyzed by Payseur et al., noticing that tetranucleotide motifs were the most variable, while trinucleotide motifs were the least variable. This analysis has been replicated in the STR size range on the census data in order to assess whether the new data would change or confirm these findings.

**Figure 7:**
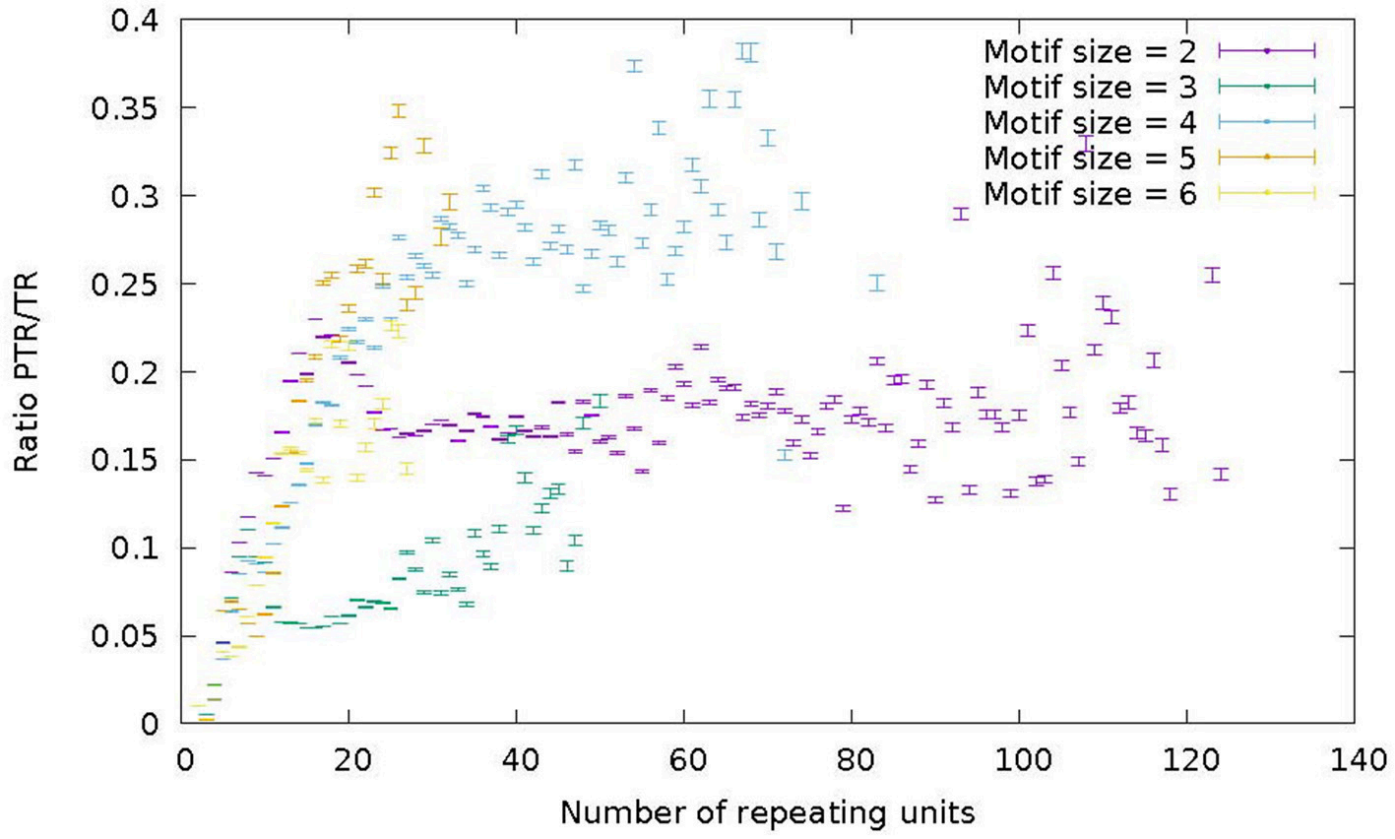
Ratio of the number of PTR over the number of candidate TR (PTR/TR ratio), subdivided in classes characterized by the size of the motif in the range 2–6 (color) and by the number of repeating units in the reference genome (abscisa). For each point in the graph the corresponding 95% Confidence Interval (CI) is given as a vertical bar.

Figure 7 represents the ratio of the number of PTR over the number of TR as a function of the number of repeat units in the reference genome. Different colors correspond to motif sizes from 2 to 6 (STR range). The ratio PTR/TR appears to be influenced by the motif size and the number of repeat units. For motif size 2 (purple) and 4 (light-blue) in Figure 7 there is a increase of the PTR/TR ratio in the range of number of repeat units from 5 to about 20, followed by a plateau in the range from about 20–60 (light blue) and 120 (purple). For motif 3 (green) and 6 (yellow) there is increase up to 20 units, followed by a fuzzy plateau up to 50 units (green) and 25 units (yellow). For motif size 5, there is a steady increase in the number of repeat units range up to 30. Each data point in the plots of Figure 7 reports also the 95% Confidence Interval estimated by bootstrapping. Also the higher variability of tetranucleotide motifs, and lower variability of trinucleotide motifs is confirmed. This trend characterized by a increase and then a leveling off is qualitatively similar to that reported by Payseur et al. (2011; Figure 1) and confirms the assertion that the higher number of repeat units in a STR correlates with a higher likelihood of observing a PTR.

**Figure 8:**
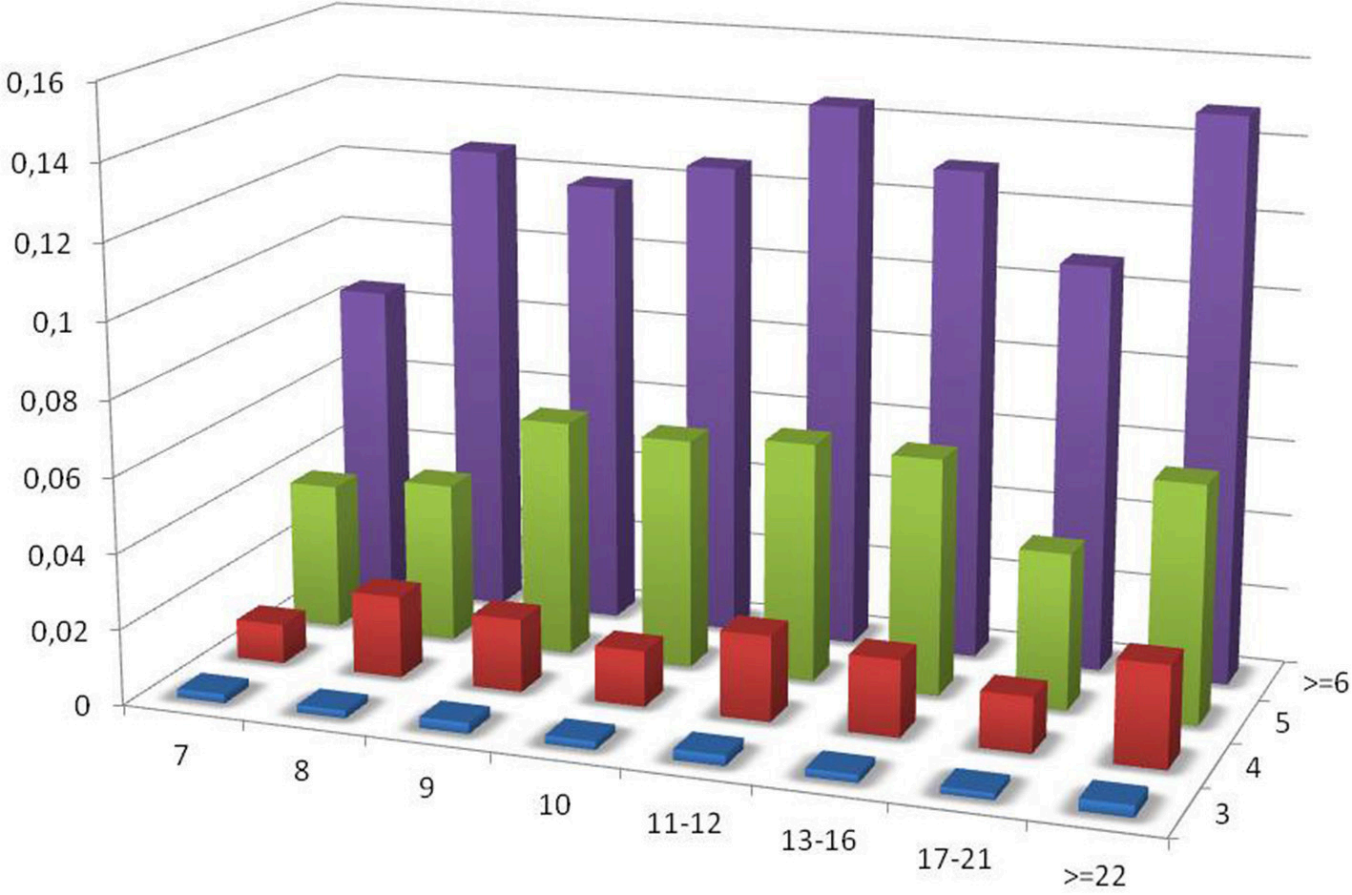
Ratio of the number of PTR over the number of candidate TR (PTR/TR ratio), subdivided in classes characterized by the size of the motif in the range above 7 (included). The vertical dimension reports the PTR/TR ratio. The width corresponds to the motif size class. The depth corresponds to the number of repeating units.

As the census contains a significant fraction of PTR in the range of VNTR, the influence of the size of the repeating unit and of the number of repeating units on the variable loci has been investigated for this sub-class. In Figure 8 the ratio PTR/TR as a function of motif size larger than 6, and of the number of repeats in the reference larger than or equal to 3 is plotted.

### 3.5. PTR/TR ratio in functional genomic locations

**Figure 9:**
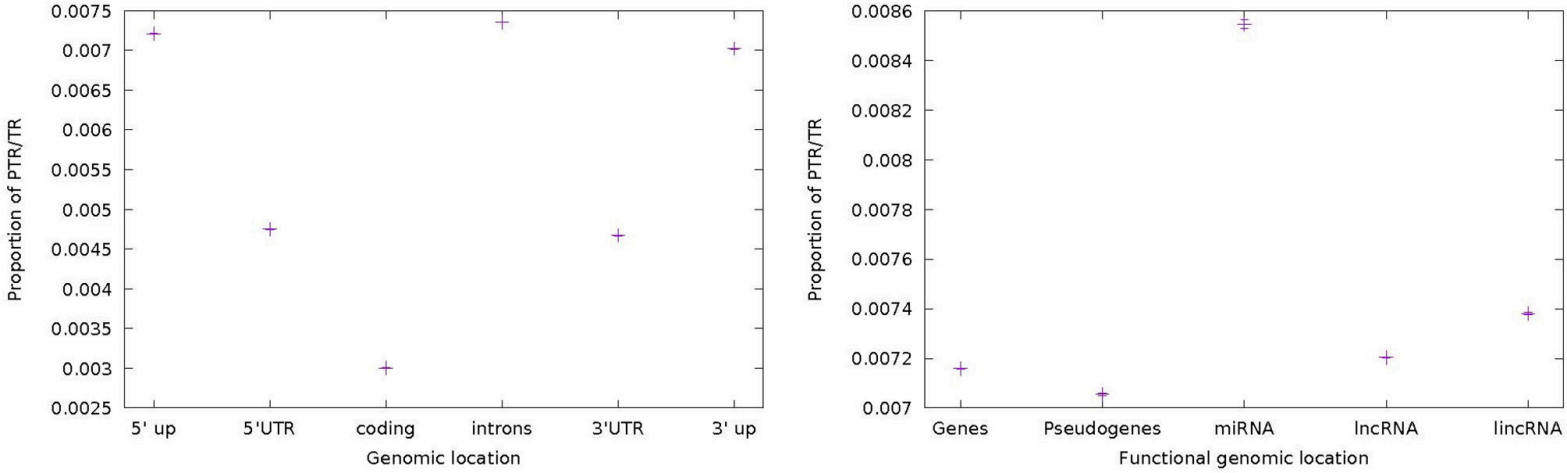
**(A)** Ratio of the number of PTR over the number of candidate (PTR/TR ratio) per genomic location among the following genomic annotations: 5′-upstream, 5′-UTR, coding, introns, 3′-UTR, 3′-upstream. **(B)** Ratio of the number of PTR over the number of candidate (PTR/TR ratio) per genomic location among the following genomic annotations: genes, pseudogenes, miRNA, lncRNA, and lincRNA. The figure shows as vertical bars also the 95% Confidence Interval estimated by bootstrapping.

Evolutionary constraints may shape TR variations differently in functionally different parts of the genome. Payseur et al. (2011; Figure 1) analyze the ratio of the number of variable microsatellites over the total number of microsatellites for the following functional regions: intergenic regions (0.028), introns (0.026), upstream from TSS (0.025), 3′-UTR, 5′-UTR (0.017) and coding exons (0.002). Our collection of PTR (and TR) has been analyzed similarly and Figure 9A reports the PTR/TR ratio for the census data: introns (0.007), 5′-upstream (0.007), 3′-upstream (0.007), 3′-UTR (0.004), 5′-UTR (0.004), and coding exons (0.003). Confidence intervals are of the order of 10^−5^. The ranking of values is in line with the fact that coding regions are overall less variable than regulatory regions and these, in turn, less variable than intronic and intragenic regions. For the coding regions our ratio is remarkably close to that in Payseur et al. (2011), while for the other regions the ratio is lower. This can be restated as the fact that increasing the number TR candidates does not result in a proportional increase in PTR in less conserved functional regions.

A second functional classification is composed of genes (0.0071), pseudogenes (0.0070), lncRNA (0.0072), lincRNA (0.0073), and miRNA (0.0085) (Figure 9B). While for the first four classes the ratio is just above 0.007, thus equivalent to that of intronic regions, it is interesting that miRNA regions have a higher ratio at 0.0085. This may point to a diffuse phenomenon of microRNA regulation mediated by TR polymorphism, such as that reported by Bandres et al. (2009). Also in this case confidence intervals are of the order of 10^−5^ and do not influence the relative ranking of the functional regions.

### 3.6. Distribution of differences in PTR loci

**Figure 10:**
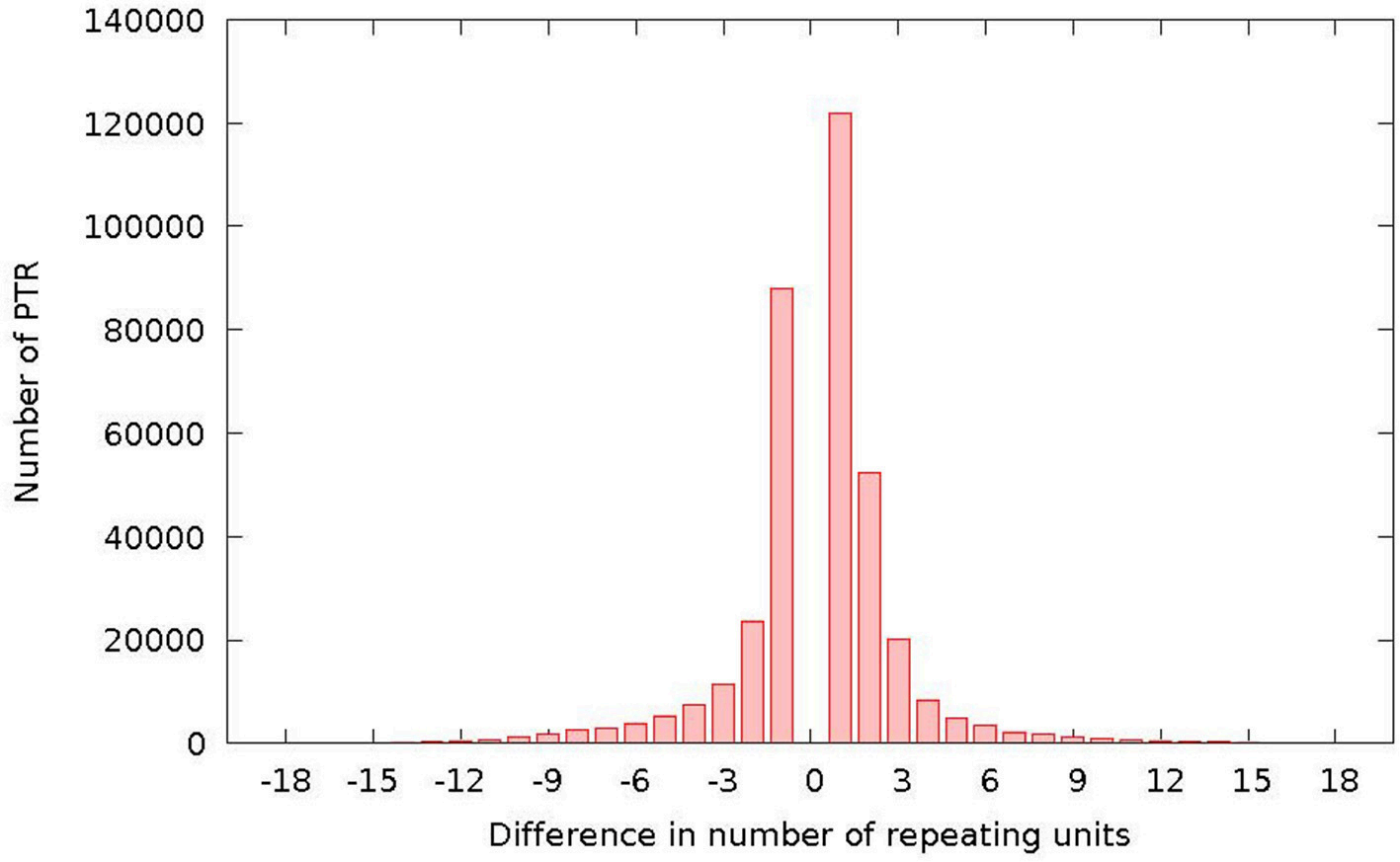
Number of detected TR polymorphisms classified as expansions (abscissa positive range) or contractions (abscissa negative range) and per difference in repeating units with respect to the same locus in the reference genome.

Figure 10 reports the distribution of the differences (in terms of motif units) between the reference and the detected polymorphism. The plot has a characteristic bell shape, with most differences concentrated in the range (−3, +3).

### 3.7. Correlations of PTR and SNP density

A question raised by Payseur et al. (2011) is whether regions with an abundant number of SNP contain also more variable microsatellite repeats. It is reported (Payseur et al., 2011) that there is a weak correlation between the two density measures (Spearman's ρ ranging from 0.06 and 0.04, with *p*-value less than 10^−15^, depending on the window size). In order to explore this issue in the context of our collection of PTR, a comprehensive list of human SNP (dbSNP release 146 from https://www.ncbi.nlm.nih.gov/projects/SNP/) was retrieved. Following a methodology described by Payseur et al. (2011) the correlation between properties of PTR and SNP density within a partition of the genome in windows of increasing size (from size 5 to 50 K bps) were analyzed by using Spearman's ρ correlation factor. Also in our data set a weak but statistically significant positive correlation (with ρ > 0.08) was measured between the *number of repeat units* of PTR (measured on the reference) and the SNP density. The analysis was done separately for the different sub-regions (3′-utr, 3′-upstream, 5′-utr, 5′-upstream, pseudogenes, introns, lncRNA, lincRNA) and the values of ρ and the *p*-values are listed in Table 3. Window size has almost no effect on the value of ρ and on the *p*-value, except for the class of PTR in long intergenic RNA where for window size 20K and 50K there is a drop to ρ = 0.078, *p* = 1.9*E* − 9.

**Table 3:** Correlation analysis of PTR number of repeat units and SNP density.

**Region**	**Spearman's ρ**	***p*-value**
3′-utr	0.104	4.56E-10
3′-upstream	0.104	3.34E-16
5′-utr	0.088	4.74E-06
5′-dowstream	0.106	2.50E-20
Pseudogenes	0.095	1.28E-16
Intronic	0.094	< 1.00E-63
lncRNA	0.087	1.22E-47
Long intergenic RNA	0.123	9.53E-26

### 3.8. Comparing the census and the landscape

In this section our catalog (aka “census”) is compared with the database compiled by Willems et al. (2014) (aka “landscape”) as a whole (differently form Sections 3.1 and 3.3 were just the clinical relevant loci were analyzed). The main differences among the two approaches were those arising from the number of genomes used to assess the polymorphic nature of a TR: 5 assembled genomes (census) vs. raw reads from the 1000 Genomes Project (landscape); and from the variety of TR detection methods used: four methods (census) vs. one (landscape). The phase of TR candidate detection and the phase of polymorphism measurement were analyzed separately.

The landscape reports on TR and PTR over the whole genome (including intergenic regions), while the census focuses on regions of interest around each gene (genic regions). In order to have a meaningful comparison, the landscape items were restricted to those that intersect genic regions.

Since the same TR may not have the exact same representation in both catalogs, some slackness was allowed in the identification procedure. A TR was represented by the set of base positions it covers, and two TR were considered to be *matching* when their Jaccard coefficient was above a threshold value *j*, for *j* = 1.0, 0.9, 0.7 and 0.5 (*j* = 1.0 corresponds to perfect identity). Since a TR in one catalog could match zero, one or more TR in the other catalog, the number of TR in one catalog with zero (no overlap), or more than zero (overlap) matches in the other catalog was considered, alternating census and landscape as source and target collection.

#### 3.8.1. TR detection phase

**Figure 11:**
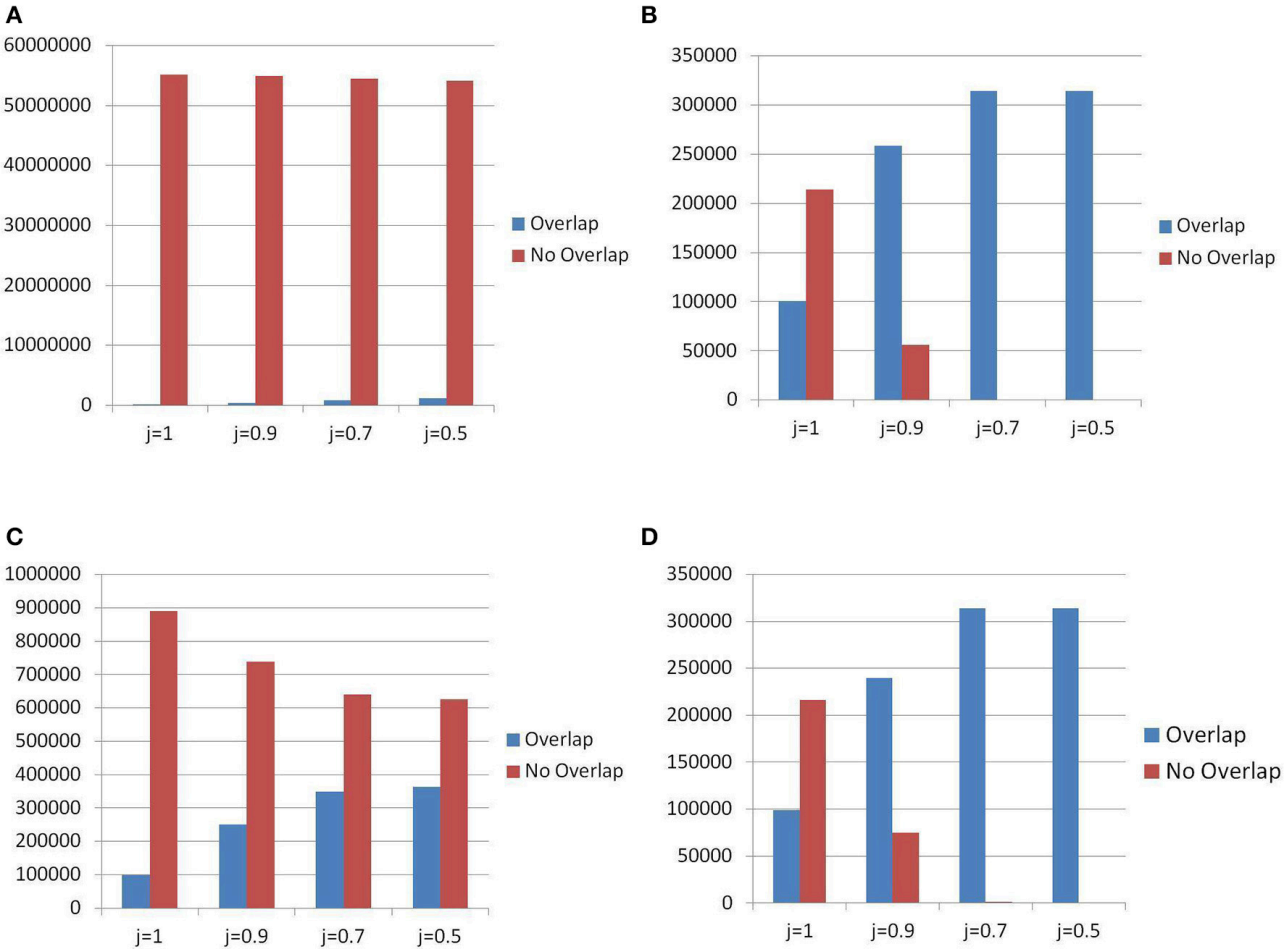
Differences in detecting candidate TR between census and landscape. In abscissa: different thresholds for the Jaccard matching formula. **(A)** All TR — Detection method for census: All Algorithms. Overlap = census items present in the landscape. No Overlap = census items not present in the landscape. **(B)** All TR — Detection method for census: All Algorithms — Overlap = landscape items present in census, No Overlap = landscape items not present in census. **(C)** All TR — Detection method for census: TRF Only — Overlap = census items present in the landscape, No Overlap = census items not present in the landscape. **(D)** All TR — Detection method for census: TRF only — Overlap = landscape items present in census, No Overlap = landscape items not present in census.

The initial listing of TR is derived for both catalogs from the reference genome (GRCh38 for the census, hg19 for the landscape converted via liftOver mapping) by applying TRF (landscape), or TRF with 3 other methods (census). See the Supplementary Materials—Appendix D for details on parameter setting and output merge operations. Thus, we expect that (almost) all TR in the landscape match a TR in the census, within the genic regions. This was indeed verified in our data (see Figure 11D), where for *j* = 0.7 about 99.5% of the landscape TR are also in the census. Figures 11B,C are almost identical. This indicates, as it was expected, that the TR in the landscape with a matching in the census are those found by applying TRF (in the census).

The census list of TR is obtained using three additional tools besides TRF, thus it was expected that many TR in the census are not present in the landscape (which has an emphasis on STR), where this difference may be attributed to our multi-tool approach, and to the lack of restriction in motif size. Indeed, Figure 11A shows that even with the slackness parameter *j* = 0.5 the vast majority of the TR in the census is not represented in the landscape.

If the effect of the additional 3 tools was remove and the class of TR found by only TRF (with similar parameters) was considered in both catalogs, the only remaining difference was in the motif size restriction, since the landscape focusses on the class of STR, while the census removes any size restriction. Figure 11C shows that (for slackness *j* = 0.5) there are many more items (almost double) in the census not reported in the landscape.

#### 3.8.2. Polymorphism measurement phase

For measuring polymorphism, the census and the landscape use two different methods: an *ad hoc* procedure (see section Methods) and lobSTR; but, even more importantly, they differ in the data used as basis for the measurement. The landscape uses raw reads data from phase 1 of the 1000 Genomes Project, thus it is able to measure not only presence/absence of polymorphism but also to give an estimate of the distribution of the TR size for each locus. However, since the raw data are made of relatively short reads (76–100 bps) there are limitations on the TR that can be analyzed in terms of motif size and total size.

**Figure 12:**
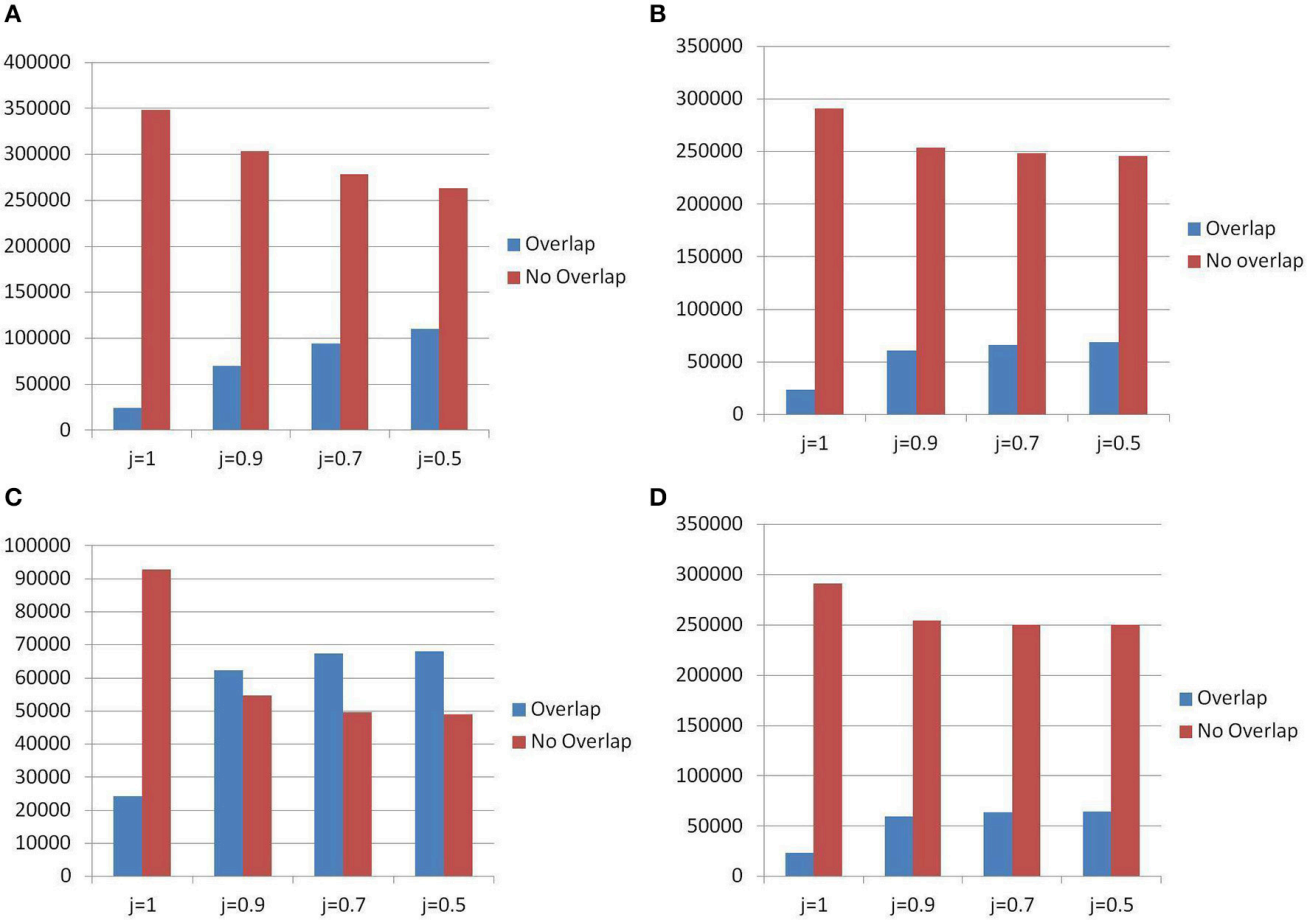
Differences in detecting PTR between census and landscape. In abscissa: different thresholds for the Jaccard matching formula. **(A)** PTR — Detection method for census: All Algorithms — Overlap = census items present in the landscape, No Overlap = census items not present in the landscape. **(B)** PTR — Detection method for census: All Algorithms — Overlap = landscape items present in census, No Overlap = landscape items not present in census. **(C)** PTR — Detection method for census: TRF Only — Overlap = census items present in the landscape, No Overlap = census items not present in the landscape. **(D)** PTR — Detection method for census: TRF only — Overlap = landscape items present in census, No Overlap = landscape items not present in census.

When the comparison was limited to those TR detected by TRF in the census and in the landscape, Figure 12C shows that (for slackness 0.9) the number of new polymorphic sites discovered in the census is about 55,000 while the number of polymorphic sites that were already certified as such in the landscape catalog is about 60,000. The additional number of 55,000 items can be attributed the extension in the range of accepted motif size.

The effect due to the larger pool of genomes from the 1000 Genomes Project (restricting to the TRF tool), was measured in Figure 12D where a ratio of 5:1 was found in favor of the landscape. Since the ratio of genomes is about 200 to 1 (1,009 vs. 5) the reduction in measurement power suffered by the census was less severe than what one could expect just based on proportions.

When also the TR found by the additional tools were considered within the census framework, Figure 12A shows that the ratio of new PTR (not registered in the landscape) to those that are confirmed polymorphic also in the landscape data is about 2.5 to 1. In particular (at threshold *j* = 0.5) about 250,000 new PTR were listed in the census. Figure 12B shows that (at threshold *j* = 0.5) about 250,000 were also missed in the census (due to the smaller number of genomes used).

### 3.9. Enrichment analysis of gene ontology classes

An enrichment analysis of the ratio of the number of PTR over the number of all TR for each of the GO categories has been done. The aim was to identify those GO categories with a statistically relevant association to a higher rate of TR polymorphism (see Supplementary Tables). By direct inspection of the top 10 positions by the Benjamini-Hochberg BH ranking, six proteins were identified, for which PTR have functional implications, or whose association to phenotypes is statistically significant, as reported in previous studies.

MUC4 (GO:0030197, extracellular matrix constituent, lubricant activity) *p*-val: 4.86135E-24, BH = 2.00287E-21, Bonf. = 2.00287E-21, fold change = 5.56. The gene MUC4 (Human mucin) was reported to be highly polymorphic, harboring numerous TR and presenting a VNTR polymorphism (Nollet et al., 1998), which might contribute to the tumorigenicity in pancreatic cancer cells (Moniaux et al., 2007).

SERPINE1 (GO:0035491, positive regulation of leukotriene production involved in inflammatory response) *p*-val = 5.98569E-17, BH = 8.22034E-15, Bonf. = 2.4661E-14, fold change = 9.27. The gene SERPINE1 has been investigated by Ju et al. (2010) to determine whether SERPINE1 intron polymorphism could affect gene expression and could be associated with diffuse-type gastric cancer susceptibility. One of the polymorphism analyzed was a VNTR and it was found to affect the gene expression levels, though it did not contribute to susceptibility.

TERT (GO:0032774, RNA biosynthetic process) *p*-val = 8.36294E-12, BH = 2.29702E-10, Bonf. = 3.44553E-09, fold change = 4.55. Concetti et al. (2013) reported that a functional VNTR of the Telomerase (TERT) gene was associated with human longevity in a population of 1,072 unrelated healthy individuals from Central Italy (18–106 years old). Jin et al. (2011) studied the impact of a functional minisatellite (MNS16A) polymorphism in the telomerase reverse transcriptase (TERT) gene on the risk of lung cancer, and on survival of patients with non-small-cell lung cancer (NSCLC), in a case/control study that consisted of 937 lung cancer patients and 943 healthy controls. This study suggested that the MNS16A VNTR polymorphism in the TERT gene has dual, conflicting, roles in lung carcinogenesis. This polymorphism might increase the risk of lung cancer development, and might also improve survival in lung cancer patients.

FAM20C (GO:0036179, osteoclast maturation) *p*-val = 9.22663E-10, BH = 2.2361E-08, Bonf. = 3.80137E-07, fold change = 3.57. Simpson et al. (2007) studied the association of lethal osteosclerotic bone dysplasia (Raine syndrome) with polymorphisms of FAM20C. A VNTR spanning an intron-exon junction of FAM20C was indicated as affecting alternative splicing.

ZP3 (GO:0001809 positive regulation of type IV hypersensitivity) *p*-val = 5.59237E-09, BH = 1.09717E-07, Bonf. = 2.30406E-06, fold change = 3.84. Chimerism analysis after allogeneic bone marrow transplantation (alloBMT) allows detection of early marrow engraftment, disease relapse, and graft rejection (Sreenan et al., 1997). Amongst other, the VNTR loci of ZP3 have been used as biomarkers of chimerism (Kletzel et al., 2013).

CD4 (GO:0006948, induction by virus of host cell-cell fusion) *p*-value = 8.49649E-09, BH = 1.53019E-07, Bonf. = 3.50055E-06, Fold change = 4.08. Salmon et al. (1993) found evidence for linkage and association between a CD4 promoter VNTR polymorphism and Type 1 diabetes mellitus (T1DM) in Danish T1DM families.

## 4. Discussion

The study of PTR (including STR and VNTR) in the human genome is gaining popularity because of several converging trends. On the one hand, more and more PTR are discovered to be associated (even causatively) with diseases (e.g., the class of repeat expansion diseases) including cancer. On the other hand, high throughput sequencing technologies are becoming instrumental in the task of measuring accurately PTR in individuals and populations, with steady technological improvements. In this context, well designed and robust catalogs of human PTR are facilitators of future disease association studies and may give insight to the global space of PTR in the human genome.

Our approach uses as input human genome assemblies and thus can be considered as a natural extension of the approach proposed by Payseur et al. (2011), that uses only one TR detection tool on the reference genome and one additional assembled genome to measure TR expansion/contraction, after positioning of the flanking regions. In the first part of the pipeline, we use four tools to uncover candidate TR in the reference genome (GRCh38), and we merge their outcome to produce an integrated candidate TR list. The use of multiple tools is novel as previous methods use only one method (mostly TRF) (Payseur et al., 2011; Gelfand et al., 2014; Willems et al., 2014). While it is not one of the purposes of our study to determine whether any single method is globally or locally superior to another, we rely on several comparative studies in the literature (e.g., Boeva et al., 2006; Leclercq et al., 2007; Pellegrini et al., 2010) showing, through *in silico* experimental evidence, that different tools uncover different TR loci, even when the comparison takes parameter setting and partial TR overlap into consideration. The second part of the pipeline is based on applications of (variants of) the classical Needleman–Wunsch alignment algorithm to measure polymorphism in five target assembled genomes after flanking region placement. This second part of the pipeline also acts as a quality filter for the list of TR, independently of the discovery tool, since a locus cannot be labeled as a high (or medium) quality PTR unless the corresponding candidate TR is also of high (medium) quality to start with (in this context, we assess alignment-based quality).

In the second part of the pipeline we identify all the TR lying on a RefSeq transcript onto five target genomes. Matching the GRCh38 transcript and the corresponding one in a target genome is done either using the NCBI remapping service (if available) or leveraging on a blast-like tool that aligns the GRCh38 sequence on the target genome. Subsequently, the flanking regions of TR are located by means of blast+ software and the sequence between them is indicated as the target TR.

A local alignment-based approach is used to assess the real presence of the TR between the flanking regions and its possible expansion/contraction.

Despite conceptually similar to that in Payseur et al. (2011), extending the above procedure to five genomes (instead of one) contributes significantly to the identification of polymorphism.

As a first release, we limited our census to the genic regions enlarged with fixed size upstream and downstream extensions since they are known to have functional roles that are likely influenced by variations in the PTR loci.

The catalog of Willems et al. (2014) is aimed at STR by using TRF on GRCh38 to define a list of candidate TR, and by applying lobSTR to the 1000 Genomes Project read data (Illumina technology), to determine polymorphism. The method of Gelfand et al. (2014) aims at discovering VNTR by applying TRF to the reference genome to define a list of candidate TR, and by applying VNTRseek to collections of reads [two trios from the 1000 Genomes Project read data (Illumina) and the Watson and Khoisan data (Roche 454 technology)], although a final catalog of VNTR is not produced. Our approach bypasses limitations due to the constraint of NGS technologies read length, and covers both STR and VNTR at the same time, by using assembled genomes as main input. The main limitation of our approach is due to the fact that there are relatively few assembled genomes in the public domain (but numbers are slowly increasing) compared to individuals sequenced with NGS. In this paper we describe a methodology and we present the final result: a catalog of 373,173 PTR in genic regions of the human genome.

As our method accepts assembled genomes as input, while lobSTR takes as input collections of reads, a validation by direct comparison of the output of the two methods on the same (digital) input is, methodologically, not well grounded. Thus we resorted to a validation via an indirect approach, cross-referencing our census with existing catalogs. We checked whether any individual sequenced in the 1000 Genome Project and in the Simon Genome Diversity Project exhibit the same polymorphism that is present in the five assembled genome we employed. We focus our analysis on the disease-oriented loci, since their polymorphic nature has been independently confirmed in literature. Sufficient comparative data were present for 26 pairs locus/polymorphism and for each such pair we could find one or more individuals in the 1KGP data set or in the SGDP data set holding the same polymorphic expansion/contraction as the one measured in the census, or within 1bp difference. Overall this test confirms the consistency of the output of our method, at a population level.

We considered several distributions of features of PTR in our catalog following the schemata of similar measures presented in the work of Payseur et al. (2011). The main purpose of these measurements is to assess whether the use of five target genomes (vs. one), the use of four tools used for candidate TR detection, and the removal of unit size restrictions (vs. focus on microsatellites) alter or confirm previous (qualitative) properties of the genomic portrait of TR variations. Overall these measures confirm previous findings, and do not reveal any odd bias in our data set.

The mean number of PTR detected per Mbp is rather uniform across the chromosomes, as it was expected. Short PTR are more abundant than long PTR, and that PTR with short repeating unit size are more abundant than those with long repeating unit size (with the notable exception of size 4 bps that is more abundant than size 3 bps).

We replicate a data analysis of Payseur et al. (2011) on the relationship between variability of loci, motif size, and number of repeating units (in the reference genome). Our analysis was performed in comparison with Payseur et al. (2011) with two objectives. This first one was to assess whether the loci reported in the census would suffer any bias, or inconsistency at a statistical level, due to the application of a novel construction pipeline. The second aim was to assess whether the trends reported by Payseur et al. for STR (Payseur et al., 2011), would be confirmed also for the class of VNTR present in the census. Although generally we measured a lower proportion of variable loci in each category, due to the abundance of candidates, the shape of our distributions are very similar to those in Payseur et al. (2011) and the same observations reported in Payseur et al. (2011) can be drawn from our data. Also, the distributions of the ratio PTR/TR are very smooth and do not present evident outliers, thus supporting our claim of robustness of the computational pipeline. Further analyses are also possible, for example Payseur et al. (2011) consider also the relative frequency of specific motif strings (of size from 2 to 6). In our case there are technical difficulties that make such analysis less cogent. As fuzzier PTR and motif of bigger size are considered, the very notion of a consensus motif become problematic (for example, a consensus string may be completely absent from the actual genomic sequence), moreover, as the number of possible strings increases exponentially with the motif size, for most classes the associated frequency quickly drops below the limit of statistical significance.

We analyze the differences in the ratio of the number of PTR over the number of candidate TR in sub-regions. Though qualitatively the relative ranking of the functional regions is similar to that noticed in Payseur et al. (2011; Figure 2), only for coding regions the value of the ratio is also roughly equal. In general in our data the ratio PTR/TR is lower than the corresponding ratio measured in Payseur et al. (2011). This is probably due to the fact that the larger pool of fuzzy TR candidates comes with diminishing returns (a smaller fraction of these candidates do result in a polymorphic behavior). This fact has been observed also by Willems et al. (2014) in the context of STR (“Shorter repeat motif, longer major allele, higher purity of the repeat motif, and residing outside of a coding region are all associated with an increase in STR variability”). We show the distribution of variations (in terms of the difference in the number of repeating units): this result is qualitatively very consistent with that reported by Payseur et al. (2011; Figure 3) and by (Willems et al., 2014, Figure 5A). The reported values of Spearman's ρ correlation coefficient between SNP and PTR density is in the range roughly from 0.08 to 0.10, with *p*-values less than 10^−10^. Thus overall the census has properties, in relation to SNP, in line with those observed in Payseur et al. (2011).

The Gene Ontology Consortium (www.geneontology.org) provides an extensive curated database of associations between genes and functional classes, for example via the “Biological Process” ontology. Thus a natural question to ask is whether a functional class (or a gene within a given functional annotation) that has a statistical significant larger ratio PTR/TR harbors abnormal expansion/contractions of TR having functional implications. This information might be used for example in order to prioritize list of genes for which further tests are scheduled when there is a hint that a malfunction may be linked to a TR expansion/contractions. For a significant threshold of 0.01 we have found 412 significant GO-classes according to the Benjamini-Hochberg corrected FDR (76 with the more stringent Bonferroni correction). We have ranked such functional classes by FDR value, and we have tested the top 10 genes contributing to the top classes in the ranking. Interestingly for six of these ten genes we could find results in literature confirming the presence of at least one PTR with a functional implication (in a wide sense). This analysis confirms on the other hand that the statistical enrichment of the ratio PTR/TR may be a sensible strategy for giving a priority to candidate genes in the context of detecting and validating PTR in functional studies.

Using as benchmark the list of 37 disease-related PTR, 18 loci are present in both the landscape and the census, 11 loci are reported only in the landscape, and 5 are listed only in the census. Uniting the two catalogs 34 (out of 37) loci are represented. An in-depth analysis of the 5 loci represented only in the census shows that these TR are often polymorphic in the SGDP data. Since the computational pipeline used in the landscape is the same of that applied to SGDP, such different behavior may be due to a the fact that SGDP data is derived from higher coverage NGS sequencing (raw data is provided in the Supplementary Materials).

Overall, fixing a Jaccard threshold to 0.5, about 100,000 PTR are present in both catalogs, about 250,000 PTR are reported only in the landscape, and about 250,000 PTR are listed only in the census. This final outcome is certainly due to several factors (number of genomes used, quality of the input data, limitations either intrinsic or imposed on the TR detection tools). From a more general point of view, both catalogs are equivalent in terms of raw numbers, when restricted to genic regions. Neither of them is complete over the whole range of PTR (including STR as well as VNTR).

One limitation of focussing on genic regions w.r.t genome-wise catalogs is that we may not report interesting PTR in inter-genic regions, having an influence on yet unknown lncRNA genes hidden in such inter-genic sequences. An extensions of our approach to handling the whole genome is planned as future research. For an exploratory applications that operates on the full spectrum of PTR, a union of the census and the landscape catalogs gives the largest set of PTR loci within human genic regions, up to date.

Although, by construction, the census is rich in long PTR of size above 100 bps (in GRCh38), it is not clear that these loci can be analyzed (e.g., by lobSTR or other similar tools) when the DNA samples are sequenced with standard Illumina NGS technology, that is characterized by a read size of a few hundred bps. The importance of the long PTR listed in the census over a longer time-frame rests on the fact that new technologies such as PacBio (Rhoads and Au, 2015), Nanopore (Magi et al., 2016) and other recently developed Single Molecule Real Time (SMRT) technologies have been found to be reliable for hard-to-sequence TR (Loomis et al., 2013; Carlson et al., 2015; McFarland et al., 2015; Liu et al., 2017) and produce much longer reads than standard NGS sequencing technologies.

Catalogs of human PTR (in particular the class of STR) are routinely used as supporting data by tools (such as lobSTR, VTNRseek, Revister) discovering PTR in collection of reads from NGS sequencing. Thus having at disposal a pool of PTR catalogs with different characteristics is a key step toward more successful exploratory association studies. Our census catalog is a valuable addition to the existing ones and is available in the public domain for the research community.

To summarize, the strong features of our catalog are: (a) inclusion of fuzzy and long PTR, with motif size beyond the size range of STR, (b) focus on gene regions, where PTR are more likely to have functional implications, and (c) about 250,000 new PTR not present in previous catalogs.

## Availability of data and material

The catalog, auxiliary data, and code are available at: http://bioalgo.iit.cnr.it/census. The catalog is given as a collection of tab separated text files (suitable for uploading and manipulations in standard sw pipelines). A file format description is provided at the above web site. The catalog data is also available as a UCSC Genome Browser Custom Track Annotation (https://genome.ucsc.edu/).

## Author contributions

MP, SD, GD, and GM exercised general supervision of the project. LG and FG conceived the main software pipeline, implemented the main procedures, and performed the statistical tests on the catalog. LC curated the compilation of the list of disease-related repeats loci. RD performed the enrichment analysis over the GO categories. EM, RB, and MS performed data collection and cross-referencing with existing catalogs, and supported the creation of the database. MP and FG wrote the manuscript and the supplementary text files. All other authors provided insight and corrections.

### Conflict of interest statement

The authors declare that the research was conducted in the absence of any commercial or financial relationships that could be construed as a potential conflict of interest.

## Footnotes

^1^See definition in the Supplementary Materials—Appendix E
