# Supplementary material for: A Census of Tandemly Repeated Polymorphic Loci in Genic Regions Through the Comparative Integration of Human Genome Assemblies
Source: Front Genet. 2018 May 2;9:155. doi: 10.3389/fgene.2018.00155 (PMC5941971; doi:10.3389/fgene.2018.00155)
Supplement: Supplementary file 1 [file Presentation_1.PDF]

frontiers

# *Supplementary Material*:
# A census of tandemly repeated polymorphic loci in genomic regions through the comparative integration of human genome assemblies

## APPENDIX A: GLOSSARY AND DEFINITIONS

- **Tandem repeats** (TR) occur in DNA when a pattern of one or more nucleotides is repeated and the repetitions are directly adjacent to each other. The repeating module is termed ”motif” or ”repeating unit”, while the number of repetitions is also called ”copy number”. For example the string ”CACACA-CACACA” is formed by the motif ”CA” repeated 6 times (but it can be interpreted also as the string ”CACA” repeated 3 times).
- **Motif**. Equivalent to ”repeating unit”. See above.
- **Copy number**. Equivalent to ”number or repetitions”. See above.
- **Pure Tandem Repeat.** When the repetitions are perfect copies of the motif, the TR is termed ‘pure’.
- **Approximate Tandem Repeat.** When a few mutations (substitutions, insertions, deletions) between repeating units are allowed, the TR it is termed ‘approximate’. There are many different mathematical models used to define Approximate Tandem Repeats depending on the metric used to measure sequence divergence among the repeating units.
- **Fuzzy Tandem Repeats** (FTR). Above a certain threshold of divergence (about 30% of mismatches, insertions and deletions) TR become harder to detect, and are termed ‘Fuzzy”. Interesting FTR must be however still be statistically distinct from background noise.
- **Short Tandem Repeats** (a.k.a. microsatellites) are TR with a motif of size (in bp) in the range between 1 and 6 bp.
- **Variable Number Tandem Repeats** (a.k.a. minisatellites) are TR with a motif of size in the range above 6 bp.
- **Variable Number of Tandem Repeats**. Equivalent to ”Variable Number Tandem Repeats”.
- **Polymorphic Tandem Repeats** are TR loci for which the copy number may be different in the genome of different individuals, without emphasis on the motif size. This class includes both STR and VNTR.
- **Jaccard coefficient**. Given two TR as intervals of genomic coordinates (in bp), their Jaccard coefficient is the ratio of the size of the intersection of the two intervals, over the size of the union of the two intervals. This number is a real number always between 0 and 1. Value 0 corresponds to completely disjoint intervals. Value 1 corresponds to completely identical intervals.

## Appendix B: Extraction of the gene sequences form ASM77258v3

The genome ASM77258v3[1] (GenBank accession GCA_000772585.3) is provided in the form of 40916 contigs assembled using the Celera Assembler v. 8.2.
At the time of this work, the NCBI remapping service did not provide a direct method to convert coordinates from ASM77258v3 to hg38. However, an indirect mapping of the gene regions can be easily derived for the large majority of sequences leveraging on the mapping form ASM77258v3 to CHM1_1. In fact, the two genomes share a large majority of the UCSC genes.
For each gene in hg38 it was checked whether it is reported in the mapping from CHM1_1 to ASM77258v3. It was also verified that the gene version is the same in both genomes. If this was the case, the coordinates for the gene in ASM77258v3 were extracted. As for the other genomes, the gene sequence was extended both upstream and downstream with 2500bps. Although in this case it was not possible to ensure that the sequences surrounding a gene would correctly map to hg38, this had no practical effect on the measure of polymorphism. Table B.1.1 reports details on the number of sequence extracted via indirect mapping.

| Genome/Mapping | Number of sequences |
|---|---|
| HG38 sequences | 104178 |
| Mapping from CHM1_1 to ASM77258v3 | 104037 |
| Intersection among all three genomes | 101239 |
| Hg38 genes not directly mapped | 2939 |
| Hg38 genes not directly mapped (Excluding ChrM) | 2918 |

**Table B.1.1 : Mapping statistics among CHM1, ASM77258v3 and HG38**

To find the coordinates of the genes for which an indirect mapping is not possible, an approach similar to the one we used for BGIAF (see Appendix C) was used. All the ASM77258v3 sequences were first aligned against hg38 obtaining the hg38 coordinates of the majority of contigs.
Then the hg38 sequences were extended with 2500bp both upstream and downstream and NCBI BLAST was used to locate the sequence on the entire ASM77258v3 genome.
Parameters were set according to the guidelines for the "assembly-assembly alignment" provided in the NCBI web site[2].
The BLAST output was sorted and filtered to exclude ambiguous alignments, and then the remaining alignments were merged into a larger one.
As for filtering, the hg38 coordinates of the contigs and the annotation of the hg38 genes were used to verify the compatibility of the alignments. In fact, if a fragment of a gene aligns on a contig whose coordinates in hg38 do not match the gene itself, then the fragment was discarded. The output of this procedure is summarized in table B.1.2.

---

[1] https://www.ncbi.nlm.nih.gov/assembly/247311
[2] http://www.ncbi.nlm.nih.gov/genome/tools/remap/docs/alignments

| Mapping | Number of sequences |
|---|---|
| Correctly mapped | 606 |
| No mapping found | 25 |
| Ambiguous mapping (unplaced) | 308 |
| Ambiguous mapping (unlocalized) | 1346 |
| Ambiguous mapping (other cases) | 633 |
| Total number of sequences | 2918 |

**Table B.1.2: Classification of the sequences not aligned using the indirect mapping**

## Appendix C: Extraction of the gene sequences form BGIAF

The genome BGIAF [1] (GenBank accession GCA_000005465.1) is provided in the form of 314786 scaffolds assembled using the SOAPdenovo v. 2010. The genome coverage is 40x.
At the time of this work, the NCBI remapping service did not provide a direct method to convert coordinates from BGIAF to any other reference genome.
Thus an ad-hoc procedure was implemented to extract from the BGIAF genome the sequences of the genes. The procedure works in three main steps.

**Phase1.** All the BGIAF sequences were first aligned against hg38. To this end the NCBI blast+ software was used following the guidelines for the "assembly-assembly alignment" provided in the NCBI web site[2]. In particular blastn was used setting the parameter perc_identity to 90. Blastn splits the input sequence in words and finds for each the best alignment. Then each word is extended until a stopping criterion is met (i.e. the alignment score drops under a predetermined threshold). As a result, a single scaffold is divided into several overlapping subsequences.

In the first phase our procedure groups all the alignments of a scaffold and applies a series of filters to verify that the scaffold properly matches on the reference and, in this case, locates it.
As a first filtering, scaffolds with not unique mapping were identified. Then the length of each alignment was compared with the length of the scaffold itself. If at least two alignments had length comparable with the entire scaffold, the scaffold was marked as "ambiguous mapping" and ignored in the rest of the procedure. For statistical purposes it was also checked whether the alignments fell on the same chromosome or not. In the first case the scaffold was reported as "unplaced" (see the NCBI website for details).
.It was considered acceptable a situation in which at most to 5% of alignments would not to belong to the most represented chromosomes. If this threshold was exceeded the scaffold was mark as "unlocalized", otherwise the discordant alignments were removed.
Subsequently the direction of the scaffold was checked. The direction of each alignment was decided by majority voting. In case of tie the scaffold was marked as "unplaced", otherwise the discordant alignments were removed. If the scaffold was reported on the minus strand it was inverted and complemented (alignments were updated accordingly).
The remaining alignments were then sorted according to the coordinates on the BGIAF genome. In absence of spurious alignments, this ordering should correspond to a ordering on HG38. When an alignment did not respect the ordering on both genomes it was removed.
A mapping of the scaffold in hg38 was computed by extracting the coordinates of the two extreme alignments.

---

[1] https://www.ncbi.nlm.nih.gov/assembly/GCA_000005465.1/
[2] http://www.ncbi.nlm.nih.gov/genome/tools/remap/docs/alignments

As a final filter, the length of the scaffold (or the mapped sub-portion) was compared with the corresponding sequence on hg38. If the size of the two sequences differed by a factor 2 or more, then the scaffold was marked as ambiguous. Table C.1.1 reports some statistics on the first phase.

| **Mapping** | **Number of scaffolds** | |
|---|---|---|
| Directly mapped | 117584 | |
| Inverted | 117895 | |
| Total Correctly mapped | | 235479 |
| Ambiguous mapping (unplaced) | 33141 | |
| Ambiguous mapping (unlocalized) | 33862 | |
| Ambiguous alignment (unlocalized) | 5876 | |
| Ambiguous direction (unplaced) | 2831 | |
| Ambiguous by size | 167 | |
| Total No mapping found | | 75877 |
| Total number of sequences | | 311356 |

**Table C.1.1: Statistics of first phase mapping of BGIAF contigs on the reference genome.**

**Phase2.** Although scaffolds, in principle, should be non-overlapping, some BGIAF sequences did cover the same position. In some cases the assembly provided contigs that were completely included in a scaffold but would cover unspecified bases (i.e. stretches of N).

When two overlapping scaffolds (or a scaffold overlapping a contig) were detected, they were merged into a single sequence and the consensus sequence was computed for the overlapping part.

The merging operation was done via a variant of the Smith–Waterman local alignment procedure between the two sequences.

During an alignment, the case when one of the sequences has an N in a given position and the other sequence has the correct nucleotide value as exploited to enhance the sequence quality. In particular, mismatches with an N are less penalized than opening a gap. Then, when rebuilding the sequence the correct nucleotide value was choosen.

For computational reasons, when the two scaffold sequences are too long (i.e. the product of their size exceeds 1Gbp) and the overlapping involves only a limited portion of them (less than 10% of the shorter sequence), the local alignment was not computed over the entire sequences. Relying on their hg-38 coordinates the overlapping portion of the strings was identified and the alignment was computed only on them.

The above procedure was repeated iteratively until no more sequences were overlapping.

The outcome of this phase was a set of 153223 non-overlapping scaffolds and the corresponding coordinates on the hg-38 reference.

**Phase3.** In the last phase the outcome of the previous phase was used to extract from BGIAF the sequences corresponding to the hg38 genes. As for the other genomes, each hg38 gene was extended of 2500 bp upstream and downstream.
The coordinates of the hg38 genes (after extending them) were intersected with the BGIAF scaffolds. In most of the cases the gene was entirely contained in a single scaffold, sometimes it was divided in two or more scaffolds (see figure C.1.2 for details). Among 104178 hg38 genes 89058 were found to be present on BGIAF (also partially).

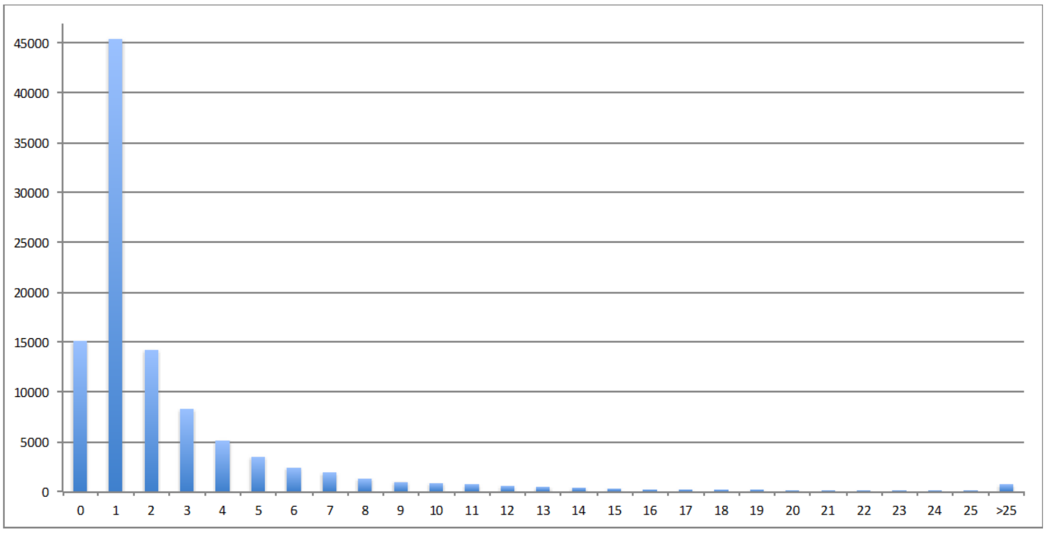


Figure C.1.2: Number of hg38 genes found in BGIAF genome stratified by number of fragments.

Then the BGIAF scaffolds associated to a gene were merged into a long single sequence filling the gap between consecutive scaffolds with a stretch of Ns as long as their genomic distance in hg38.
This sequence was subsequently aligned with that of the gene in hg38 to find the exact endpoints of the gene.
Again, BLAST+ was used for this latter alignment and a filtering strategy similar to that described in the first phase to compute the endpoints.

# Appendix D. Parameter Settings for TR Finding Tools.

## D.1. Parameter setting

### *Hardware configuration*

Our experiments were run on a dual Intel Xeon processor (32 cores) at 3.1GHz, with 64Gb RAM memory. The Operating System was Mac OS X 10.8.5. The HD holds 4Tb.

### *Software repositories*

A stand-alone copy of all the software was requested to the authors or downloaded from the authors' webpages.

| Software name | Version | URL |
|---|---|---|
| TRF | 4.07b | https://tandem.bu.edu/trf/trf.html |
| MREPS | 2.5 | http://mreps.univ-mlv.fr/ |
| Tandem SWAN | N/A | http://favorov.bioinfolab.net/swan/tool.html |
| TrStalker | 3.0 | http://bioalgo.iit.cnr.it:8080/treads[1] |

### *Software parameters*

**TRF:** TRF was run using two sets of parameters and the results were merged into a single list. The first set of parameters derives from the TRF settings used by Willems at al. [WGH+14], while the second set of parameters was taken from Gelgand et al. [GHLB14]. In both articles TRF was used to find variable tandem repeats in collections of reads from high-throughput sequencing data. A comprehensive description of the parameters and their meaning can be found in the author's website: https://tandem.bu.edu/trf/trf.unix.help.html

Command line (1st run): `trf407b.macos64 sequence.fa 2 7 7 80 10 50 500 -d –h`
where: the match score was set to 2, the mismatch score was set to 7, the delta (indel) score was set to 7, the PM score was 80, the PI score was 10, the minimum score of a tandem repeat to be reported was 50, the maximum period of a reported TR was 500.

Command line (2nd run): `trf407b.macos64 sequence.fa 2 5 7 80 10 50 2000 -d –h`
where: the match score was set to 2, the mismatch score was set to 5, the delta (indel) score was set to 7, the PM score was 80, the PI score was 10, the minimum score of a tandem repeat to be reported was 50, the maximum period of a reported TR was 2000.

**mreps:** mreps has two main parameters to control the degree of fuzziness of tandem repeats: res and exp. A tutorial about mreps parameters can be found in the authors' website: http://mreps.univ-mlv.fr/tutorial.html

Command line: `mreps.macosx.bin -res 5 -exp 3.0 -fasta sequence.fa`
where:

[1] Web interface for the TrStalker software. The original command line executable has been provided by the authors.

- The resolution parameter depends on the TR period. According to the authors: "*For small periods (up to 10-15), resolution 5 is usually sufficient to find all meaningful repeats*". Since all of the TRs in our benchmark datasets had small period the default setting –res 5 was used
- The exponent is the ratio between the period and the TR length (i.e. the number of copies). As default the authors set it to 3.

**swan:** tandemSWAN parameters are mostly devoted to limit the size of the reported repeats ( -L controls the Minimal Length of Repeats, -u controls the repeat unit size upper limit –l controls the repeat unit size lower limit). Since the default values did match our evaluation datasets, they were left unchanged.
TandemSWAN has also an important parameter allowing filtering according to the statistical significance of the TR. It enables two mutually exclusive modes: Motifs and MaSK (see the authors' website for details). The default mode is MaSK. Since this mode appears to be more appropriate for tandem repeats it was used it in our evaluation.

The comprehensive description of the tandemSWAN parameters can be found in the author's website: http://favorov.bioinfolab.net/swan/options.html

Command line: `swan -f sequence.fa -F 1`

where the parameter –F 1 indicates that the input is in fasta format.
Note that tandemSWAN was not available under MacOS, thus the code was executed on an Ubuntu Linux virtual machine installed in the same, above-mentioned, hardware architecture.

**TrStalker:** this software has been designed to match many possible task requirements: from protein repeats identification to tandem repeats in DNA. This versatility has the cost of the need of setting several parameters that are specified via configuration file. The software authors have provided a configuration file tailored on tandem repeats for DNA sequences.

```
#### remove TRs included in other TRs (yes/no) ####
removeCovered = yes
#### similarity between patterns ####
similarity = 0.7
#### coefficient for similarity between adjacent copies ####
adSim = 1.5
#### number of following probes to take into account: used only by
TRStalker ####
numSubsequentProbes = 5
#### threshold multiplier####
thresholdmultiplier = 3.0
#### tha maximum value admitted for threshold ####
saturation = 0.7
#### the sequence is split into bulks of this dimension ####
maxLen = 2000
#### bulks overlap ####
maxRepeatedRegionLen = 1000
#### repeat type: Simple (STR) or Neighbouring (NTR) ####
trdefinition = STR
#### minimum pattern length to take into account ####
lungMin = 3
```

```
#### most popular pattern hit length ####
NUM_TOP_DISTANCES = 50
### number of processors
processors = 4
### edit or score (levenshtein/weighted)  ###
scoreFunction = levenshtein
```

Command line: `java -jar RunnablePtrStalker.jar -c TRStalkerV3.config -i sequence.fa -v 3 -o outfile.txt`
where the parameter –v 3 indicated to use the version 3 (latest) of the software.

## D.2. Brief description of the key ideas  distinguishing the tools

*TRF (Tandem Repeat Finder)*

This is the most broadly used software. Its definition of tandem repeat is based on $n$-independent Bernulli trials. Interestingly TRF explicitly models the probability of match/mismatch and the probability of insertions and deletions between two consecutive copies of the repeated sequence. This software works in two phases: detection and analysis. The first phase is aimed at selecting a set of candidate tandem repeats, the second step refines the output filtering out irrelevant repeats and collecting a series of statistics about the retained results. During detection the algorithm assumes that, according to the Bernulli model, adjacent copies must contain a certain number of matching characters in the corresponding positions. Given a small integer $k$, the algorithm computes all the possible $\Sigma^k$ words in the alphabet $\Sigma$ ($\Sigma = \{A, C, G, T\}$). The input sequence is scanned and the position of each word instance in the sequence is annotated. The distance between consecutive instances of a given word is used as a guess of the period length. As all seed based aligning algorithm, TRF's success depends on the presence of the seed in the majority of the copies of the repeating unit. Once a candidate pattern has passed the detection stage, it is aligned with the surrounding sequence by means of a dynamic programming algorithm. To limit the computational cost of this phase, an approximate method (narrow band alignment) is used. Lastly, TRF further refine the alignment computing (via average voting) a consensus sequence and use it to realign the tandem repeat.

*mreps*

The objective of this software is to capture maximal tandem repeats allowing fractional copy number. This choice is biologically motivated and it helps to reduce artefacts such as finding pairs of tandem repeats whose coordinates differ for just one position. The algorithm works in two phases: one (called upper frame) where a combinatorial algorithm is used to find candidate tandem repeat sequences, another (named lower frame) where a heuristic is used to retain only biologically relevant results. The algorithm adopts the *maximal* run as definition of tandem repeat. Let $p$ be an integer and $S$ a string, a substring $s'$ is a maximal run if for each substring of length $2p$ in $s'$ the number of mismatches between the characters in position $i$ and $i + p$ is 0 and it is different from 0 for the strings partially overlapping $s'$. This definition can be extended to consider impure sequences allowing a certain number of mismatches. The authors use a result from the literature to compute these maximal runs. In the second step, the authors employ ad-hoc heuristics to: trim edges of the tandem repeats (when mismatches are accumulated in the extreme positions), and cope with duplicates differing only for the

period length. The lower frame completes after applying a final relevance filtering where too small and too noisy tandem repeats are discarded.

### *TandemSWAN*

This software allows controlling the degree of fuzziness of the returned tandem repeats. The key idea behind this method is that of measuring local properties of a sequence that hold only in presence of a tandem repeat. In particular let $T$ be the hypothesized period and let $c_i$ be the character in position $i$ in the sequence. In presence of a tandem repeat it is more likely that $c_i = c_{i-T}$ and $c_i = c_{i+T}$. Let $s_i$ count the number of mismatches among $c_i$ and its neighbours at distance $T$, in presence of a tandem repeat of length $T$, the sum $S[i] = s_i + \cdots s_{i+T-1}$ is minimal. TandemSWAN computes this summation for each position in the input sequence and for each possible $T$ in a user-defined range. When $S[i]$ drops under a certain threshold the position $i$ is marked as a candidate starting position of a tandem repeat of period $T$. The list of candidate repeats is filtered so that to eliminate overlapping among results. Filtering is based on two mutually exclusive statistical models of significance, namely: MaSK and Motif. The first model is the default choice running the software and better fits our dataset. According to MaSK, tandemSWAN computes combinatorial properties of the candidate repeats and returns the element that minimize them. The most important property in MaSK mode is the minimal number of identical symbols in corresponding repeat positions.

### *TrStalker*

This software has been designed with the goal of finding fuzzy tandem repeats (namely tandem repeats with a low purity or a large divergence from the consensus motif and the other instances). The algorithm adopts the edit distance as a metric to compare sequences. TrStalker works in phases. As a first step the algorithm attempts to guess the period of a candidate tandem repeat. The authors observe that computing q-grams over a random sequence embedding a tandem repeat of period $k$, the frequency of duplicate q-grams of length $k$ is statistically much higher than the others. In order to deal with mismatches in the sequence, the authors employ gapped q-grams that allow the use of a certain number of unspecified characters. Instead of filtering, TrStalker uses a ranking function to sort candidate length values to be verified. The output of the first step is a pair indicating the candidate starting position and period. Subsequently the algorithm verifies the presence of a tandem repeat in the candidate locus. To do this, TrStalker uses a dynamic programming algorithm to align the candidate tandem repeat with a generalized median string. Lastly the algorithm post-processes the output by removing results completely enclosed in other repeats.

## Appendix E: Repeat Expansion Diseases

| | Disease | **Gene** | **Reference** |
|---|---|---|---|
| 1 | Huntington (HD) | Huntingtin (*HTT*) | Loureiro JR, Oliveira CL, Silveira I. 2016 Unstable repeat expansions in neurodegenerative diseases: nucleocytoplasmic transport emerges on the scene. Neurobiol Aging. **39**:174-83. |
| 2 | Huntington 2 (HDL2) | Junctophilin-3 (*JPH3*) | Loureiro JR et (2016) |
| 3 | Spinocerebellar ataxia 1 (SCA1 ) | Ataxin-1 (*ATXN1*) | Loureiro JR et (2016) |
| 4 | Spinocerebellar ataxia 2 (SCA2) | Ataxin-2 (*ATXN2*) | Loureiro JR et (2016) |
| 5 | Spinocerebellar ataxia 3 (SCA3) | Ataxin-3 (*ATXN3*) | Loureiro JR et (2016) |
| 6 | Spinocerebellar ataxia 6 (SCA6 ) | P/Q Ca ++ Channel (*CACNA1A*) | Loureiro JR et (2016) |
| 7 | Spinocerebellar ataxia 7 (SCA7) | Ataxin-7 (*ATXN7*) | Loureiro JR et (2016) |
| 8 | Spinocerebellar ataxia 8 (SCA8) | Ataxin-8 (*ATXN8*) | Loureiro JR et (2016) |
| 9 | Spinocerebellar ataxia 12 (SCA12) | Protein Phosphatase 2, Regulatory Subunit B, BETA (*PPP2R2B*) | Loureiro JR et (2016) |
| 10 | Spinocerebellar ataxia 17 (SCA 17) | TATA-binding protein (*TBP*) | Loureiro JR et (2016) |
| 11 | Spinocerebellar ataxia 10 (SCA10) | Ataxin-10 (*ATXN10*) | Loureiro JR et (2016) |
| 12 | Friedreich Ataxia | Frataxin (*FXN*) | Loureiro JR et (2016) |
| 13 | Spinal and Bulbar Muscular Atrophy (SBMA) – Kennedy disease | Androgen Receptor (*AR1*) | Loureiro JR et (2016) |
| 14 | Dentatorubral-Pallidoluysian Atrophy (DRPLA) | Atrophin-1 (*ATN1*) | Loureiro JR et (2016) |
| 15 | Fragile X | Fragile X mental retardation 1 (*FMR1*) | Loureiro JR et (2016) |
| 16 | Mental retardation, X-linked, FRAXE type (FRAXE) | AF4/FMR2 Family Member 2 (*AFF2*) | Loureiro JR et (2016) |
| 17 | Myotonic Dystrophy type 1 (DM1) | Dystrophia Myotonic Protein Kinase (*DMPK*) | Loureiro JR et (2016) |
| 18 | Myotonic Dystrophy type 2 (DM2)/ Proximal Myotonic Myopathy (PROMM) | Zinc Finger Protein 9 (*ZNF9*) | Loureiro JR et (2016) |
| 19 | Amyotrophic lateral Sclerosis/ Frontotemporal Dementia (ALS-FTD 2) | Chromosome 9 Open Reading Frame 72 (*C9orf72*) | Loureiro JR et (2016) |
| 20 | Schizophrenia | Potassium Calcium-Activated Channel Subfamily N Member 3 (*KCNN3*) | Grube S, Gerchen MF, Adamcio B, Pardo LA, Martin S, Malzahn D, Papiol S, Begemann M, Ribbe K, Friedrichs H, et al 2011. A CAG repeat polymorphism of KCNN3 predicts SK3 channel function and cognitive performance in schizophrenia. EMBO Mol Med **3(6):** 309–319. |
| 21 | Male Infertility | DNA Polymerase | Rovio AT1, Marchington DR, Donat S, Schuppe |

| | | | |
|---|---|---|---|
| | | Gamma, Catalytic Subunit (*POLG1*) | HC, Abel J, Fritsche E, Elliott DJ, Laippala P, Ahola AL, McNay D, et al 2001. Mutations at the mitochondrial DNA polymerase (POLG) locus associated with male infertility. Nat Genet. **29(3):**261-2. |
| 22 | Congenital Hypoventilation Syndrome | Achaete-Scute Family BHLH Transcription Factor 1 (*ASH-1*) | de Pontual L1, Népote V, Attié-Bitach T, Al Halabiah H, Trang H, Elghouzzi V, Levacher B, Benihoud K, Augé J, Faure C, et al 2003 Noradrenergic neuronal development is impaired by mutation of the proneural HASH-1 gene in congenital central hypoventilation syndrome (Ondine's curse).Hum Mol Genet. **12(23):**3173-80. |
| 23 | Baltic Myoclonus | Cystatin B(*CSTB*) | Virtaneva K1, D'Amato E, Miao J, Koskiniemi M, Norio R, Avanzini G, Franceschetti S, Michelucci R, Tassinari CA, Omer S, et al 1997.Unstable minisatellite expansion causing recessively inherited myoclonus epilepsy, EPM1. Nat Genet. **15(4):**393-6. |
| 24 | Creutzfeldt-Jakob disease | Prion Protein (*PRPN*) | Kovacs GG, Trabattoni G, Hainfellner JA, Ironside JW, Knight RS, Budka H, 2002 Mutations of the prion protein gene phenotypic spectrum. J Neurol **249**:1567–1582 |
| 25 | Spinocerebellar ataxia 36 (SCA36) | NOP56 Ribonucleopro tein (*NOP56*) | Kobayashi H, Abe K, Matsuura T, Ikeda Y, Hitomi T, Akechi Y, Habu T, Liu W, Okuda H, Koizumi A, 2011. Expansion of intronic GGCCTG hexanucleotide repeat in NOP56 causes SCA36, a type of spinocerebellar ataxia accompanied by motor neuron involvement. Am J Hum Genet. ;**89(1**):121-30. doi: 10.1016/j.ajhg.2011.05.015. |
| 26/27 | Fuchs Corneal Dystrophy | Transcription Factor 4 (*TCF4*) | Wieben ED, Aleff RA, Tosakulwong N, Butz ML, Highsmith WE, Edwards AO, Baratz KH, 1997 .A common trinucleotide repeat expansion within the transcription factor 4 (TCF4, E2-2) gene predicts Fuchs corneal dystrophy. Proc Natl Acad Sci U S A. **94(14):**7458-63. |
| 28 | Congenital Hypoventilation Syndrome | Paired Like Homeobox 2b (*PHOX2B*) | Brown LY, Brown SA. Alanine tracts: the expanding story of human illness and trinucleotide repeats, 2004. Trends Genet;**20(1**):51-8. |
| 29 | Infantile spasm syndrome | Aristaless Related Homeobox (*ARX*) | Brown LY et al (2004) |
| 30 | Hand-Foot-Genital syndrome | Homeobox A13 (*HOXA13*) | Brown LY et al (2004) |
| 31 | Oculophayngeal Muscular Dystrophy | Poly(A) Binding Protein Nuclear 1 (*PABPN1*) | Brown LY et al (2004) |
| 32 | Holomprosencephaly | Zic Family Member 2(Zic2) | Brown LY et al (2004) |
| 33 | Synpolydactyly | Homeobox D13(*HOXD13*) | Brown LY et al (2004) |
| 34 | Mental Retardation X-Linked with growth hormone deficiency | SRY-Box 3 (*SOX3*) | Brown LY et al (2004) |
| 35/36 | Cleidocranial dysplasia | Runt Related Transcription Factor 2 (*RUNX2*) | Brown LY et al (2004) |
| 37 | Blepharophimosi Ptosis epicanthus inversus syndrome | Forkhead Box L2 (*FOXL2*) | Brown LY et al (2004) |
